# A Facile Synthesis for Novel Loperamide Analogs as Potential μ Opioid Receptor Agonists

**DOI:** 10.3390/molecules171214288

**Published:** 2012-12-03

**Authors:** Xiaofeng Bao, Duliang Liu, Yanyan Jin, Yao Yang

**Affiliations:** Department of Biochemical Engineering, Nanjing University Science & Technology, Chemical Engineering Building B302, 200 Xiaolinwei, Nanjing 210094, China; E-Mails: ldl12358@hotmail.com (D.L.); j.yy.1002c@163.com (Y.J.); bxfeng11@hotmail.com (Y.Y.)

**Keywords:** loperamide analogs, μ opioid receptor agonist, morphine, opioid ligand receptors

## Abstract

A facile synthesis for novel loperamide analogs as potential μ opioid receptors is described. The synthetic procedure for compound **5**, which contains two 4-phenyl piperidine scaffolds, was optimized, and this compound was synthesized in excellent yield. We also describe a mild and highly efficient protocol for the synthesis of compounds **6** and **7**.

## 1. Introduction

**Figure 1:**
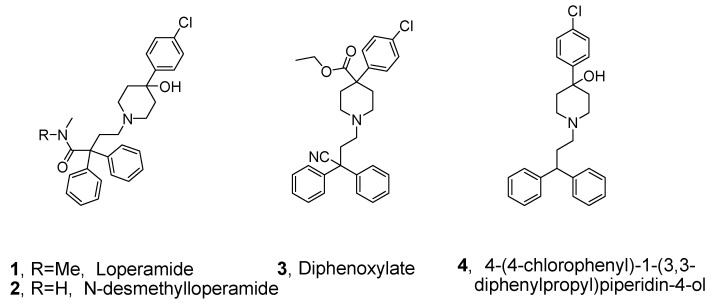
Structures of representative MOR agonists.

Opioid ligand receptors are involved in various physiological activities, including analgesia, miosis, bradycardia, general sedation, hypothermia, insensitivity and depression of the flexor reflexes and have been widely used in medicine, most prominently in the treatment of pain [1,2]. Three separate receptors—μ (mu), δ (delta), and κ (kappa)—were proven to be the basis of the pharmacologic responses using *in vitro* radioligand binding affinity assays and *in vivo* localization of labeled drug in tissue homogenates or sections [3,4]. These reports indicate that μ opioid receptor (MOR) agonists are useful analgesics in the periphery, especially for inflamed tissues [5,6]. Phenylpiperidine structures, such as loperamide (**1**) [7,8], *N*-desmethylloperamide (**2**) [9], diphenoxylate (**3**) [10], and **4** (Figure 1) [11], are typical representatives of these compounds. Although diphenoxylate and loperamide are MOR agonists and show affinity and selectivity for the cloned μ human opioid receptor, they do not easily pass through the blood–brain barrier (BBB) [12], therefore, they are currently mainly used as antidiarrheal drugs.

More recently, MOR agonists based on the 4-phenylpiperidine scaffold have been reported and have shown promising results [13,14]. The therapeutic potential of loperamide and other compounds based on 4-phenylpiperidine scaffolds may also be of interest to the scientific community in light of the recent discovery of DiPOA, a novel, systemically available and peripherally restricted MOR agonist with antihyperalgesic activity [15].

**Figure 2:**
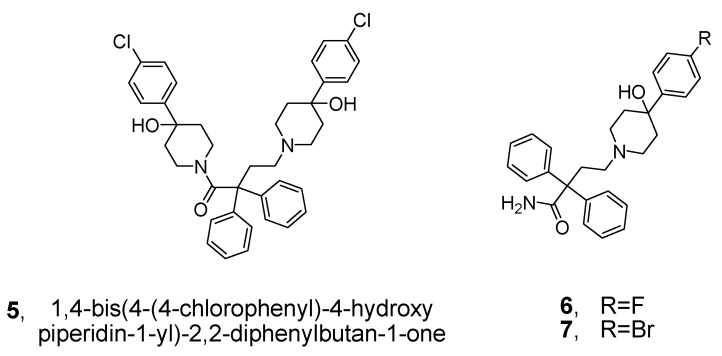
Structures of novel loperamide analogs.

Therefore, the compounds 1,4-bis(4-(4-chlorophenyl)-4-hydroxypiperidin-1-yl)-2,2-diphenylbutan-1-one (**5**, Figure 2), 4-(4-(4-fluorophenyl)-4-hydroxypiperidin-1-yl)-2,2-diphenylbutanamide (**6**, Figure 2) and (*Z*)-2-(2-(4-(4-fluorophenyl)-4-hydroxypiperidin-1-yl) ethyl)-3-methylene-2-phenylhex-4-enamide (**7**, Figure 2), which are based on loperamide and diphenoxylate, were designed and synthesized as potential MOR agonists. The novel analog **5** contains two 4-phenylpiperidine scaffolds, and analogs **6** and **7** each contain one 4-phenylpiperidine scaffold. We consider these analogs to be promising MOR agonists.

## 2. Results and Discussion

As a consequence of our interest in MORs containing 4-phenylpiperidine scaffolds, we developed a mild and highly efficient protocol for the synthesis of compounds **5**, **6** and **7**. We initiated our studies by preparing the key intermediate **10b** by coupling commercially available compounds **8** and **9b** to achieve product **10b** under different reaction conditions, as shown in Table 1. The reaction using conditions based on those of a similar reaction that was previously reported in the literature was not successful for an unknown reason [16]. When the reaction time was prolonged to 30 h with 3 eq. of DIPEA at 105 °C, we successfully isolated compound **10b** in a disappointing 6% yield. The possible reasons for failure may be that the hydroxyl group on compound **9b** was affected. This group is also a strong nucleophile under basic conditions, as observed in our previously published study [17], and the carboxylates present under basic conditions may also compete with the secondary amine in the nucleophilic substitution reaction. Because the overall yield of **10b** using this approach was poor, an alternate strategy was adopted for synthesizing a high yield of **10b**.

**Scheme 1:**
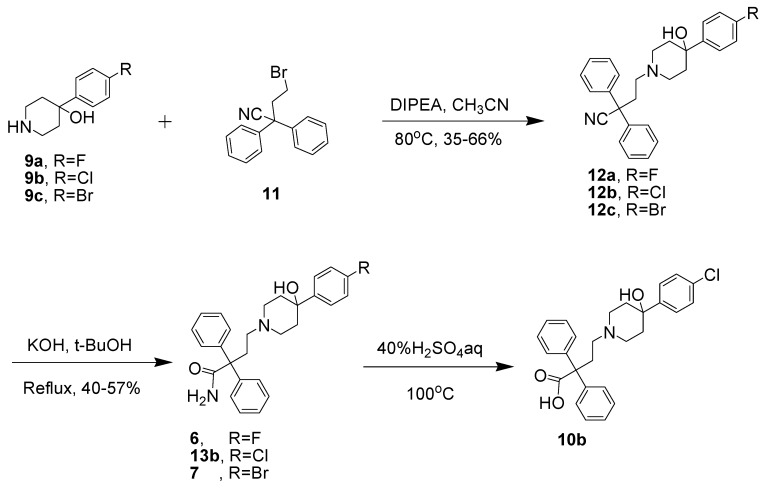
Synthesis of the amides **6**, **13b** and **7**.

Subsequently, we designed another approach to prepare the intermediate **10b** involving the alkylation of 4-(4-chlorophenyl)-4-hydroxylpiperidine with 4-bromo-2,2-diphenylbutyronitrile in the presence of DIPEA, followed by the hydrolysis of **12b** with KOH in ^t^BuOH to afford compound **13b** (Scheme 1) [18]. We also prepared analogs **6** and **7** using a similar procedure for the future radiolabeling study. The slow hydrolysis of amide precursor **13b** in 40% H_2_SO_4_ aq. at reflux gave a trace amount of **10b**. The hydrolysis of nitrile compound **12b** under different reaction conditions was also investigated (Table 2). Compound **10b** was obtained in 7% yield when the reaction was catalyzed with H_2_O_2_/NaOH in H_2_O at 80 °C for 20 h [19,20]. A trace amount of **10b** was obtained when an inorganic base (2 M sodium hydroxide) was used as a catalyst [21]. We isolated two byproducts, **14b** (20%~23% yield) and **15b** (66%~72% yield), when the reaction was performed under acidic conditions (37% HCl or 40% H_2_SO_4_) [22]. The principal reason was that the tertiary hydroxyl group on **13b** was very easily eliminated under acidic conditions to form an alkene [17].

**Scheme 2:**
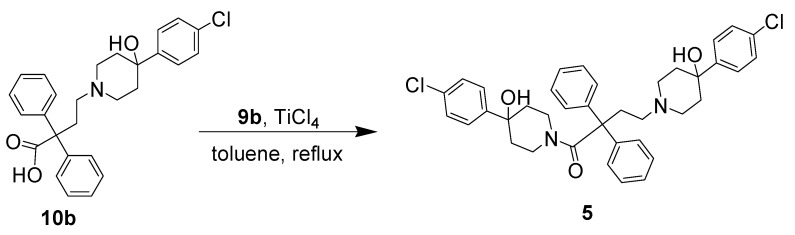
Synthesis of compound **5**.

The desired product **5** was obtained in low yield by coupling **10b** with the amine **9b** in a reaction catalyzed by TiCl_4_ in toluene refluxing for 20 h (Scheme 2) [23]. Unfortunately, this second approach was not feasible for use in the rest of the study.

**Scheme 3:**
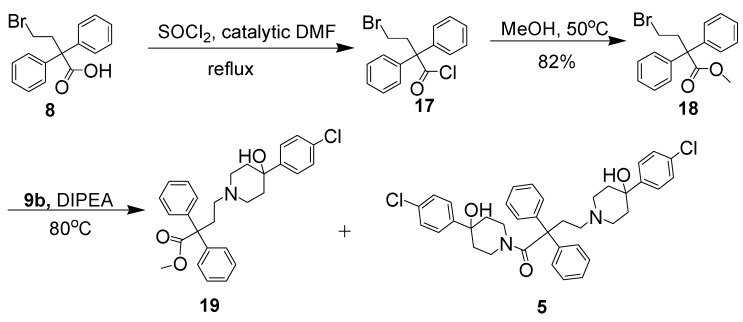
The Synthesis of the ester **19** and **5**.

When the first approach was analyzed in detail, it was determined that the formation of a substantial amount of salt at the start of reaction may be responsible for the low yield of **12b**. Thus, a new method involving the protection of the carboxyl group of **8** with a methyl group to form an ester precursor before coupling with **9b** was developed (Scheme 3). A mixture of 4-bromo-2,2-diphenylbutyric acid **8**, thionyl chloride and catalytic DMF in dichloromethane was stirred and refluxed for 3 h. After concentration under reduced pressure, crude 4-bromo-2,2-diphenylbutyric acid chloride (**17**) was achieved as a pale yellow oil. Compound **17** was converted into the ester **18** in excellent yield (82%) by reaction with methanol at 50 °C for 4 h. The desired product, **5**, was obtained as a white solid in 71% yield by condensing **18** with **9b** in the presence of DIPEA in CH_3_CN at 80 °C for 15 h (Scheme 3). The side product **19** was also obtained as a pale orange solid in 8% yield.

## 3. Experimental

### 3.1. General

All reagents and organic solvents were ACS grade or higher and used without further purification. Unless otherwise noted, all chemicals were purchased from J&K Scientific (Shanghai, China). Reactions were performed under argon atmosphere with standard Schlenk techniques. Thin layer chromatography was performed on HAIYANG silica gel F254 plate, and compounds were visualized under UV light (λ = 254 nm). Column chromatography was carried out using HAIYANG silica gel (type: 200–300 mesh ZCX-2). ^1^H (500 MHz), ^13^C-NMR (126 MHz) and ^19^F-NMR (470 MHz) spectra were recorded on an Avance 500 spectrometer (Bruker; Billerica, MA, USA). Chemical shifts are reported in δ units (ppm) downfield relative to the chemical shift for tetramethylsilane.

*4-(4-(4-Chlorophenyl)-4-hydroxypiperidin-1-yl)-2,2-diphenylbutanoic acid* (**10b**). *Entry 2.* The 4-bromo-2,2-diphenylbutanoic acid **8** (200 mg, 0.6266 mmol) was dissolved in THF (10 mL), followed by addition of 4-(4-chlorophenyl)-piperidin-4-ol (133 mg, 0.6266 mmol, 1 eq.) and DIPEA (0.242 mL, 1.8798 mmol, 3 eq.) to the solution. The mixture was refluxed for 2 days. No new product was found via TLC. *Entry 5*. The 4-bromo-2,2-diphenylbutanoic acid **8** (400 mg, 1.253 mmol) was suspended in acetonitrile (10 mL) and DIPEA (0.675 mL, 3.75 mmol) was added. The mixture was stirred at 105 °C for 30 h. After the solvent was removed under vacuum, the crude was dissolved in CH_2_Cl_2_ and was introduced onto a silica gel column. The product was eluted with 3% MeOH in dichloromethane to give **10b** as a pale-orange solid (20 mg, 6% yield). TLC (silica gel; MeOH–CH_2_Cl_2_ (5:95 v:v); R_f_ = 0.40). LC-MS (M^+^+1): found 449.88; calcd for C_27_H_29_ClNO_3_, 450.18.

*4-(4-(4-Chlorophenyl)-4-hydroxypiperidin-1-yl)-2,2-diphenylbutanenitrile* (**12b**). 4-(4-Chlorophenyl)-4-hydroxypiperidine (2.1 g, 10.0 mmol) was suspended in acetonitrile (15 mL) and DIPEA (3.5 mL, 30 mmol) was added. 4-Bromo-2,2-diphenylbutanenitrile (3.00 g, 10 mmol) in acetonitrile (15 mL) was then added. The reaction mixture was stirred at 70 °C for 30 h. After the solvent was removed under vacuum, the crude material was redissolved in CH_2_Cl_2_ and introduced onto a silica gel column. The product was eluted with 5% MeOH in dichloromethane to yield **12b** as a pale-orange solid (2.8 g, 66% yield). TLC (silica gel; MeOH–CH_2_Cl_2_ (5:95 v:v); R_f_ = 0.50). ^1^H-NMR (CDCl_3_): δ 7.37 (m, 14H), 2.80 (d, 2H, *J* = 11.25 Hz), 2.67 (m, 2H), 2.55 (m, 2H), 2.47 (t, 2H, *J* = 11.3 Hz), 2.105 (m, 2H), 1.73 (d, 2H, *J* = 11.9 Hz). ^13^C-NMR (CDCl3): *δ* 140.16, 132.95, 129.09, 128.57, 128.10, 126.95, 126.23, 122.27, 71.11, 54.93, 50.17, 49.71, 38.52, 36.80ppm. LC-MS (M^+^+1) found 431.20; calcd for C_27_H_28_ClN_2_O, 431.19.

*4-(4-(4-Fluorophenyl)-4-hydroxypiperidin-1-yl)-2,2-diphenylbutanenitrile* (**12a**). Compound **12a** was synthesized using the same procedure that was followed the synthesis of the compound **12b**, and was obtained in 35% yield as a pale-orange solid. ^1^H-NMR (CDCl_3_): δ 7.45 (m, 2H), 7.39 (d, 4H, *J* = 10 Hz), 7.35 (t, 4H, *J* = 5Hz ), 7.29 (t, 2H, *J* = 10 Hz ), 7.00 (t, 2H, *J* = 10 Hz), 2.75 (d, 2H, *J* = 10 Hz), 2.63 (m, 2H), 2.52 (m, 2H), 2.47 (t, 2H, *J* = 10 Hz), 2.08 (m, 2H), 1.71 (d, 2H, *J* = 15 Hz).^13^C-NMR: δ 162.89, 160.93, 144.27, 140.16, 129.03, 128.05, 126.91, 126.45, 126.39, 122.23, 115.18, 115.01, 70.91, 54.88, 50.19, 49.72, 38.56, 36.74. ^19^F-NMR: δ 116.12. LC-MS (M^+^+1): Found: 415.06; Calcd: C_27_H_28_FN_2_O: 415.22.

*4-(4-(4-Bromophenyl)-4-hydroxypiperidin-1-yl)-2,2-diphenylbutanenitrile* (**12c**). Compound **12c** was synthesized using the same procedure that was followed the synthesis of the compound **12b**, and was obtained in 58% yield as a pale-orange solid. ^1^H-NMR (CDCl_3_): δ 7.48 (d, 2H, *J* = 10 Hz), 7.41 (d, 4H, *J* = 10 Hz), 7.37 (m, 6H), 7.31 (t, 2H, *J* = 5 Hz), 2.76 (d, 2H, *J* = 10 Hz), 2.65 (m, 2H), 2.54 (m, 2H), 2.45 (t, 2H, *J* = 10 Hz), 2.08 (m, 2H), 1.68 (m, 3H). ^13^C-NMR: δ 147.64, 140.11, 131.47, 129.07, 128.09, 126.91, 126.66, 122.25, 121.00, 70.97, 54.90, 50.18, 49.64, 38.35, 36.67. LC-MS (M^+^+1): Found: 476.92; Calcd: C_27_H_28_BrN_2_O: 477.13.

*4-(4-(4-Chlorophenyl)-4-hydroxypiperidin-1-yl)-2,2-diphenylbutanamide* (**13b**). 4-(4-(4-Chloro-phenyl)-4-hydroxypiperidin-1-yl)-2,2-diphenylbutanenitrile (2.5 g, 6.0 mmol) was dissolved in BuOH (40 mL) and potassium hydroxide (1.2 g, 21.3 mmol) was added. The reaction mixture was stirred at 100 °C for 3d. After concentration under vacuum, the crude material was redissolved in dichloromethane and filtered through a pad of Celite. Chromatography of the sample on a silica gel column eluted with ammonium hydroxide (2 M) solution in MeOH-CH_2_Cl_2_ (5:95 v/v) gave **13b** as a pale-yellow solid (1.58 g, 55% yield). TLC (silica gel; MeOH–CH_2_Cl_2_ (5:95 v:v); R_f_ = 0.40). ^1^H-NMR (CDCl_3_): δ 7.36 (m, 14H), 6.58 (s, 1H), 5.46 (s, 1H), 3.91 (s, 1H), 2.83 (m, 2H), 2.69 (s, 2H), 2.39 (m, 3H), 2.10 (m, 2H), 1.74 (d, 2H, *J* = 12.85 Hz). ^13^C-NMR: 176.56, 167.25, 158.96, 146.67, 143.26, 132.82, 128.69, 128.41, 127.06, 126.10, 59.91, 54.92, 49.48, 38.28, 35.87. LC-MS (M^+^+1): Found: 449.3; Calcd: C_27_H_30_ClN_2_O_2_: 449.20.

*4-(4-(4-Fluorophenyl)-4-hydroxypiperidin-1-yl)-2,2-diphenylbutanamide* (**6**). Compound **6** was synthesized using the same procedure that was followed the synthesis of the compound **13b**, and was obtained in 40% yield as a pale-orange solid. ^1^H-NMR (CDCl_3_): δ 7.45 (m, 2H), 7.31 (m, 10H), 6.99 (t, 2H, *J* = 10 Hz), 5.07 (s, 1H), 2.89 (m, 2H), 2.64 (m, 2H), 2.35 (m, 2H), 2.04 (m, 2H), 1.59 (d, 2H, *J* = 5 Hz), 6.22 (s, 1H), 5.82 (s, 1H), 3.02 (m, 2H), 2.82 (m, 4H), 2.63 (m, 2H), 2.36 (m, 2H), 1.76 (d, 2H, *J* = 10 Hz). ^13^C-NMR: δ 175.53, 162.43, 160.51, 145.88, 143.83, 129.27, 128.46, 127.29, 127.24, 127.07, 115.15, 114.98, 69.28, 59.54, 54.73, 49.44, 37.33, 29.55. ^19^F-NMR: δ 117.01.

*4-(4-(4-Bromophenyl)-4-hydroxypiperidin-1-yl)-2,2-diphenylbutanamide* (**7**). Compound **7** was synthesized using the same procedure that was followed the synthesis of the compound **13b**, and was obtained in 57% yield as a pale-orange solid.^1^H-NMR (CDCl_3_): δ 7.41 (t, 2H, *J* = 5 Hz), 7.31 (m, 12H), 6.45 (s, 1H), 5.87 (s, 1H), 2.82 (m, 12H), 2.31 (m, 12H), 2.50 (m, 12H), 2.42 (m, 12H), 2.11 (m, 12H), 1.69 (m, 12H). ^13^C-NMR: δ 176.79, 147.17, 143.14, 131.46, 128.75, 128.57, 127.26, 126.64, 121.06, 70.67, 59.88, 55.00, 49.48, 37.79, 35.46. LC-MS (M^+^+1): Found: 494.93; Calcd: C_27_H_30_BrN_2_O_2_^+^: 495.14.

*4-(4-(4-Chlorophenyl)-4-hydroxypiperidin-1-yl)-2,2-diphenylbutanoic acid* (**10b**). 4-(4-(4-Chloro-phenyl)-4-hydroxycyclohexyl)-2,2-diphenylbutanamide (1.2 g, 2.6 mmol) was added to 40%H_2_SO_4_ (50 mL) and the mixture was stirred at 100 °C for 2 days. The crude material was extracted with ethyl acetate (60 mL×3). The ethyl acetate was removed by reduced pressure distillation. After drying with MgSO_4_ and dissolving in CH_2_Cl_2_ (2 mL), the product was introduced onto a silica gel and eluted with 5% MeOH in CH_2_Cl_2_ to give trance amount of **10b**. TLC (silica gel; MeOH–CH_2_Cl_2_ (5:95 v:v); R_f_ = 0.30). ^1^H-NMR (CDCl_3_): δ 7.3096(m, 14H), 5.9319 (s, 1H), 3.29 (m, 2H), 2.94 (m, 2H), 2.80 (m, 2H), 2.72 (m, 2H), 2.67 (m, 4H), 2.10 (m, 2H). LC-MS (M^+^+1): found: 449.88; Calcd for C_27_H_28_ClNO_3_^+^, 450.18.

*Procedure for Preparing* **10b** *from* **12b**

*Entry 3*. 4-(4-(4-Chlorophenyl)-4-hydroxypiperidine-1-yl)-2,2-diphenylbutanenitrile (400 mg, 0.928 mmol) was dissolved in 1,4-dioxane (10 mL) and 37% hydrochloric acid (5 mL) was then added. The reaction mixture was refluxed for 16 h. After concentration under vacuum, the crude was dissolved in trichloromethane (10 mL) and extracted with H_2_O (20 mL × 3). After the organic layer was dried by MgSO_4_, the solvent was removed under vacuum and redissolved in CH_2_Cl_2_. The mixture was introduced onto a silica gel column and the product was eluted with 50% EtOAc in hexane first and 5% MeOH in CH_2_Cl_2_ later to give the product **14b** as a yellow solid (250 mg, 66%). TLC (silica gel; EtOAc–hexane (1:1 v:v); R_f_ = 0.70). Compound 14b: ^1^H-NMR (CDCl_3_): δ 7.36 (m, 14H), 6.04 (m, 1H), 3.16 (m, 2H), 2.74 (m, 2H), 2.62 (m, 4H), 2.53 (m, 2H) ppm. ^13^C-NMR (CDCl_3_): δ 141.44, 140.57, 135.56, 134.26, 130.53, 129.92, 129.55, 128.27, 127.72, 123.52, 55.96, 54.81, 51.93, 51.57, 38.48, 29.44 ppm. MS (M^+^+1): found 412.99; Calcd C_27_H_25_ClN_2_^+^, 412.95. 

*1,4-bis(4-(4-Chlorophenyl)-4-hydroxypiperidin-1-yl)-2,2-diphenylbutan-1-one* (**5**). 4-(4-Chloro-phenyl)-4-hydroxypiperidinorophenyl)-4-hydroxypiperidine (28 mg, 0.12 mmol, 2 eq.) was dissolved in toluene (5 mL) and 4-(4-(4-chlorophenyl)-4- hydroxypiperidin-1-yl)-2,2-diphenylbutanoic acid (30 mg, 0.067 mmol) and TiCl_4_ (1.5 mL) was then added. The mixture was refluxed for 20 h. After the solvent was removed under vacuum, the crude material was dissolved in CH_2_Cl_2_ (10 mL). After the mixture was filtered and CH_2_Cl_2_ was then removed to 2 mL, the product was introduced onto a silica gel with 5% MeOH in CH_2_Cl_2_ to give 5 as a pale-orange solid (trace amount). TLC (silica gel column; MeOH–CH_2_Cl_2_ (5:95 v:v); R_f_ = 0.40).

*Methyl 4-bromo-2,2-diphenylbutanoate* (**18**). 4-Bromo-2,2-diphenylbutanoic acid (2.0 g, 6.266 mmol) was dissolved in dry CH_2_Cl_2_ (20 mL) and thionyl chloride (2.27 mL, 31.33 mmol, 5 eq.) was then added slowly. A trace amount of DMF was added later. The reaction mixture was refluxed at N_2_ for 3 h. Methyl alcohol (2 mL, excess) was added slowly and the mixture was stirred at 50 °C for 3 h. After the solvent was removed under vacuum, the crude material was redissolved in CH_2_Cl_2_ and introduced onto a silica gel column. The product was eluted with 10% EA in hexane to give a pale-orange oil (900 mg, 43% yield).TLC (silica gel; EtAc–hexane (10:90 v:v); R_f_ = 0.80). ^1^H-NMR (CDCl_3_): δ 7.31 (m, 10H), 3.74 (s, 1H), 3.13 (m, 2H), 3.00 (m, 2H).^13^C-NMR (CDCl_3_): δ 174.05, 141.70, 128.70, 128.42, 127.46, 60.84, 52.78, 42.0447, 29.18 ppm. GC-MS: found: 331.99; Calcd for C_17_H_17_BrO_2_, 332.04.

*Methyl4-(4-(4-chlorophenyl)-4-hydroxypiperidin-1-yl)-2,2-diphenylbutanoate* (**19**) *and 1,4-bis(4-(4-chlorophenyl)-4-hydroxypiperidin-1-yl)-2,2-diphenyl -butan-1-one* (**5**). Methyl 4-bromo-2,2-diphenylbutanoate (800 mg, 2.4 mmol) was dissolved in CH_3_CN (15 mL) and 4-(4-chlorophenyl)-4-hydroxypiperidine (406 mg, 1.92 mmol, 0.8 eq.) was then added. The reaction mixture was stirred at 50 °C for 30 h. After concentration under vacuum, the crude material was redissolved in CH_2_Cl_2_ and introduced onto a silica gel column. The product was eluted with 3% MeOH in CH_2_Cl_2_ first and 5% MeOH in CH_2_Cl_2_ later to get **19** as a pale-orange solid (70 mmg, 8% yield) and 20 as a pale-orange solid (437 mg, 71%). Compound **19**: TLC (silica gel; MeOH-CH_2_Cl_2_ (10:90 v:v); R_f_ = 0.40). ^1^H-NMR (CDCl_3_): δ 7.34 (m, 14H), 3.74 (s, 3H), 3.28 (2H, m), 3.13 (2H, m), 3.12 (2H, m), 3.10 (4H, m), 2.60 (1H, s), 2.06 (2H, d, J = 14.0 Hz). ^13^C-NMR (CDCl_3_): δ 173.06, 143.62, 140.18, 132.54, 127.72, 127.52, 127.28, 126.62, 126.32, 125.04, 68.58, 58.02, 51.81, 48.22, 34.51, 31.73, 30.93, 28.36, 21.69. MS: found: 463.92; calcd for C_28_H_31_ClNO_3_^+^, 464.20. Compound **5**: TLC (silica gel; MeOH–CH_2_Cl_2_ (10:90 v:v); R_f_ = 0.40). ^1^H-NMR (CDCl_3_): δ 7.3094 (m, 18H), 4.5945 (d, 1H, J = 11.4 Hz), 3.31 (m, 10H), 2.73 (m, 2H), 2.55 (m, 2H), 2.28 (m, 2H), 1.87 (m, 4H), 1.65 (m, 2H). ^13^C-NMR (CDCl_3_): 171.38, 146.46, 145.29, 141.45, 136.64, 133.00, 132.68, 129.45, 125.03, 128.89, 128.45, 128.31, 128.17, 127.57, 126.54, 126.08, 71.10, 69.06, 60.03, 54.44, 44.25, 42.68, 39.65, 39.21, 37.91, 35.95, 35.30, 29.77 ppm. MS: found: 644.88; Calcd for C_38_H_4__2_Cl_2_N_2_O_3_^+^, 644.25.

## 4. Conclusions

In summary, compound **5** was synthesized in excellent yield. Analogs **6** and **7** were obtained through the hydrolysis of the nitrile group in good yield. These methods have the advantages of high yields, convenient procedures, and mild reaction conditions.

## Figures and Tables

**Table 1:**
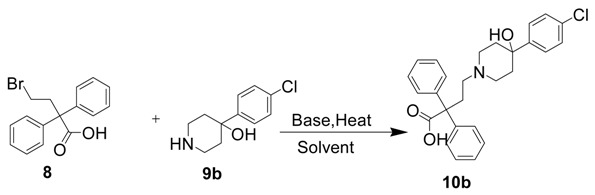
Synthesis of the intermediate compound **10b**.

Entry	Ratio 8:9b:DIPEA	Reaction conditions	Yield *10b
1	1:1.5:0	THF, reflux, 2 days	-
2	1:1:3	THF, reflux, 2 days	-
3	1:1:3	CH_3_CN, 70 °C, 30 h	-
4	1:1:3	CH_3_CN, 85 °C, 30 h	Trace
5	1:1:3	CH_3_CN, 105 °C, 30 h	6%

* Isolated yield.

**Table 2:**
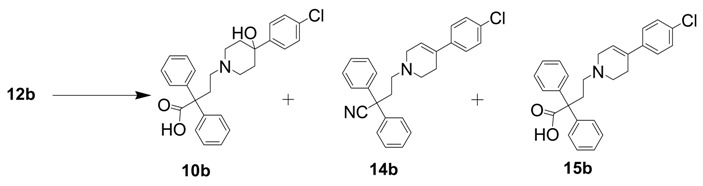
Hydrolysis of the nitrile **12b**.

Entry	Catalyst	Reaction conditions	Yield (%) *		
			10b	15b	14b
1	NaOH ^a^	CH_3_OH/H_2_O, Reflux, 30 h	trace	-	-
2	NaOH ^a^	H_2_O, Reflux, 30 h	trace	-	-
3	HCl ^b^	1,4-Dioxane, Reflux, 16 h	-	20	66
4	H_2_SO_4_^c^	H_2_O, 100 °C, 16 h	-	23	72
5	H_2_O_2_^d^/NaOH ^a^	H_2_O, 80 h, 20 h	7	-	-

Catalyst concentration: ^a^ 2 M; ^b^ 37%; ^c^ 40%; ^d^ 30%. * Isolated yield.

## Supplementary Materials

Supplementary data associated with this article can be found in the online version (^1^H-NMR, ^13^C-NMR, and LC-MS for all compounds), which can be accessed at: http://www.mdpi.com/1420-3049/17/12/14288/s1.
