# Supplementary material for: A Facile Synthesis for Novel Loperamide Analogs as Potential μ Opioid Receptor Agonists
Source: Molecules. 2012 Dec 3;17(12):14288–97. doi: 10.3390/molecules171214288 (PMC6268526; doi:10.3390/molecules171214288)

Supporting Information

Table of Contents

^{1}H-NMR, ^{13}C-NMR and MS Spectrum of all compounds.

Figure S1. 500 MHz ^{1}H-NMR spectrum of Compound **10b**. **S3**

Figure S2. MS spectrum of Compound **10b**. **S4**

Figure S3. 500 MHz ^{1}H-NMR spectrum of Compound **12a**. **S5**

Figure S4. 125 MHz ^{13}C-NMR spectrum of Compound **12a**. **S6**

Figure S5. 470 MHz ^{19}F-NMR spectrum of Compound **12a**. **S7**

Figure S6. MS spectrum of Compound **12a**. **S8**

Figure S7. 500 MHz ^{1}H-NMR spectrum of Compound **12b**. **S9**

Figure S8. 500 MHz ^{1}H-NMR spectrum of Compound **12c**. **S10**

Figure S9. 125 MHz ^{13}C-NMR spectrum of Compound **12c**. **S11**

Figure S10. MS spectrum of Compound **12c**. **S12**

Figure S11. 500 MHz ^{1}H-NMR spectrum of Compound **6**. **S13**

Figure S12. 125 MHz ^{13}C-NMR spectrum of Compound **6**. **S14**

Figure S13. ^{19}F-NMR spectrum of Compound **6**. **S15**

Figure S14. 500 MHz ^{1}H-NMR spectrum of Compound **13b.** **S16**

Figure S15. 100 MHz ^{13}C-NMR spectrum of Compound **13b**. **S17**

Figure S16. LC-MS spectrum of Compound **13b**. **S18**

Figure S17. 500 MHz ^{1}H-NMR spectrum of Compound **7.** **S19**

Figure S18. 125 MHz ^{13}C-NMR spectrum of Compound **7**. **S20**

Figure S19. MS spectrum of Compound **7**. **S21**

Figure S20. 500 MHz ^{1}H-NMR spectrum of Compound **14b**. **S22**

Figure S21. 125 MHz ^{13}C-NMR spectrum of Compound **14b**. **S23**

Figure S22. MS spectrum of Compound **14b**. **S24**

Figure S23. 500 MHz ^{1}H-NMR spectrum of Compound **18**. **S25**

Figure S24. 125 MHz ^{13}C-NMR spectrum of Compound **18**. **S26**

Figure S25. MS spectrum of Compound **18**. **S27**

Figure S26. 500 MHz ^{1}H-NMR spectra of Compound **19**. **S28**

Figure S27. 125 MHz ^{13}C-NMR spectrum of Compound **19**. **S29**

Figure S28. MS spectrum of Compound **19**. **S30**

Figure S29. 500 MHz ^{1}H-NMR spectrum of Compound **5**. **S31**

Figure S30. 125 MHz ^{13}C-NMR spectrum of Compound **5**. **S32**

Figure S31. MS spectrum of Compound **5**. **S33**

The Spectrum of Compounds.

Figure S1. 500 MHz ^{1}H-NMR spectrum of Compound **10b**.

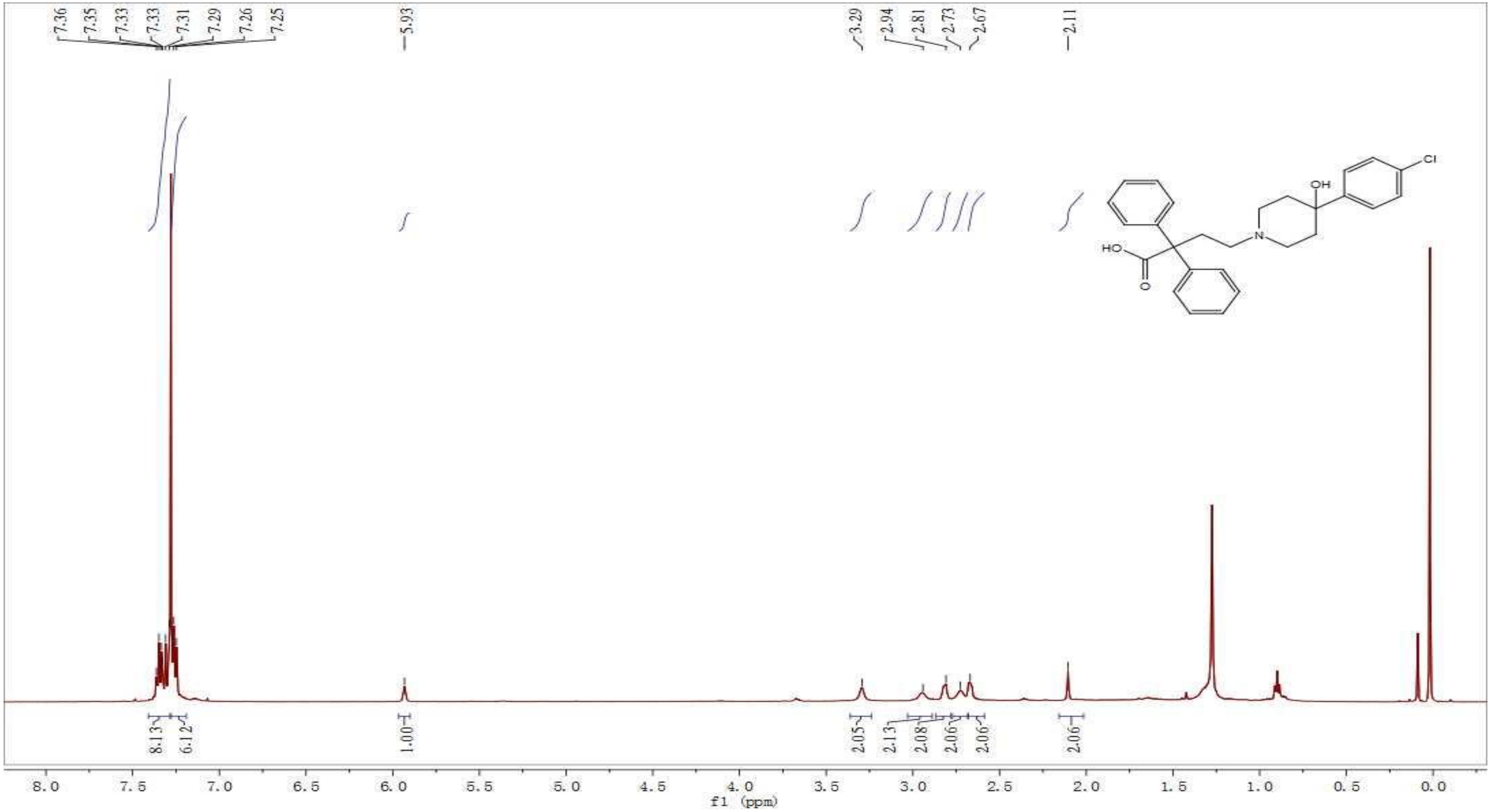

Figure S2. MS spectrum of Compound **10b**.

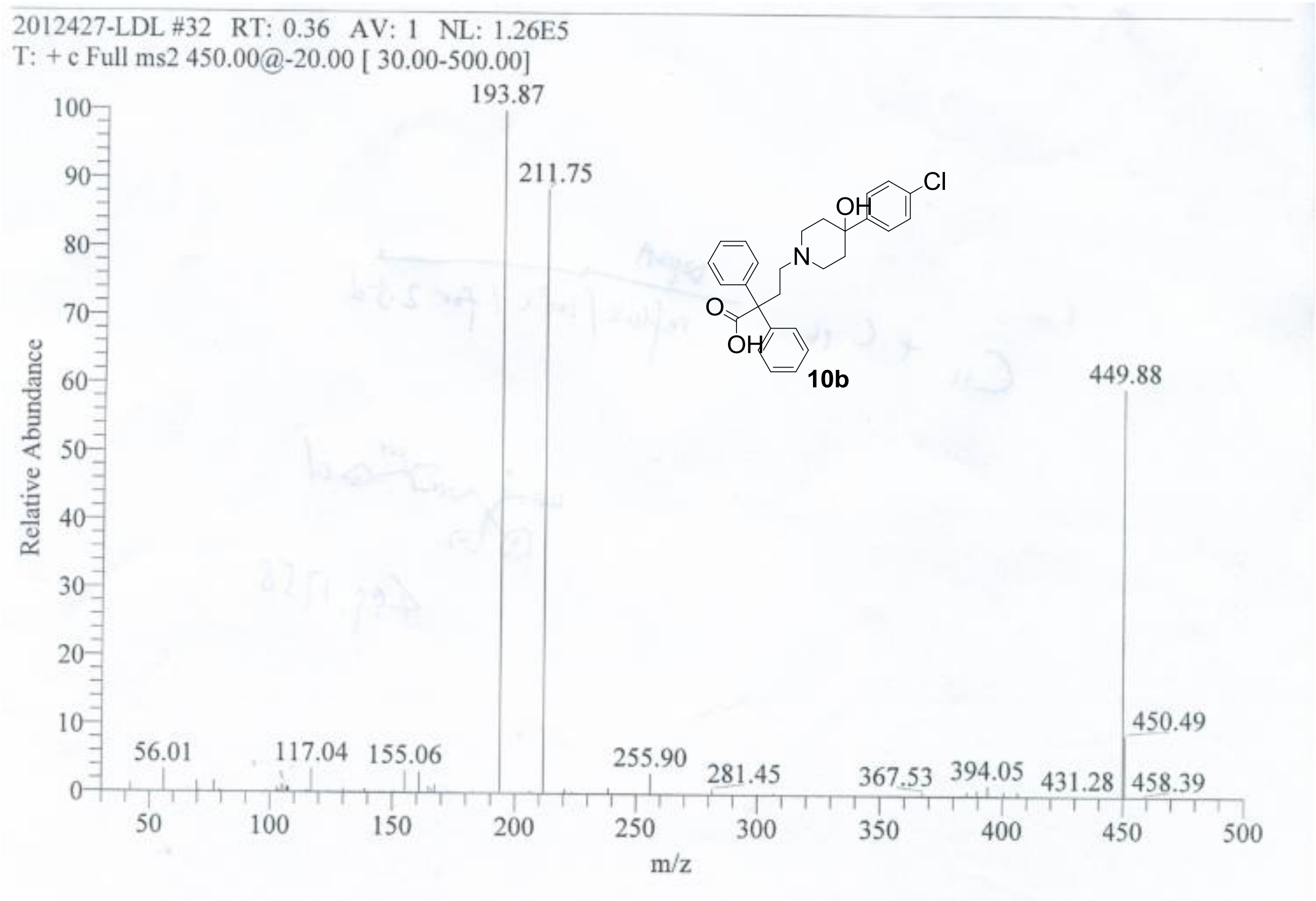

Figure S3. 500 MHz ^{1}H-NMR spectrum of Compound **12a**.

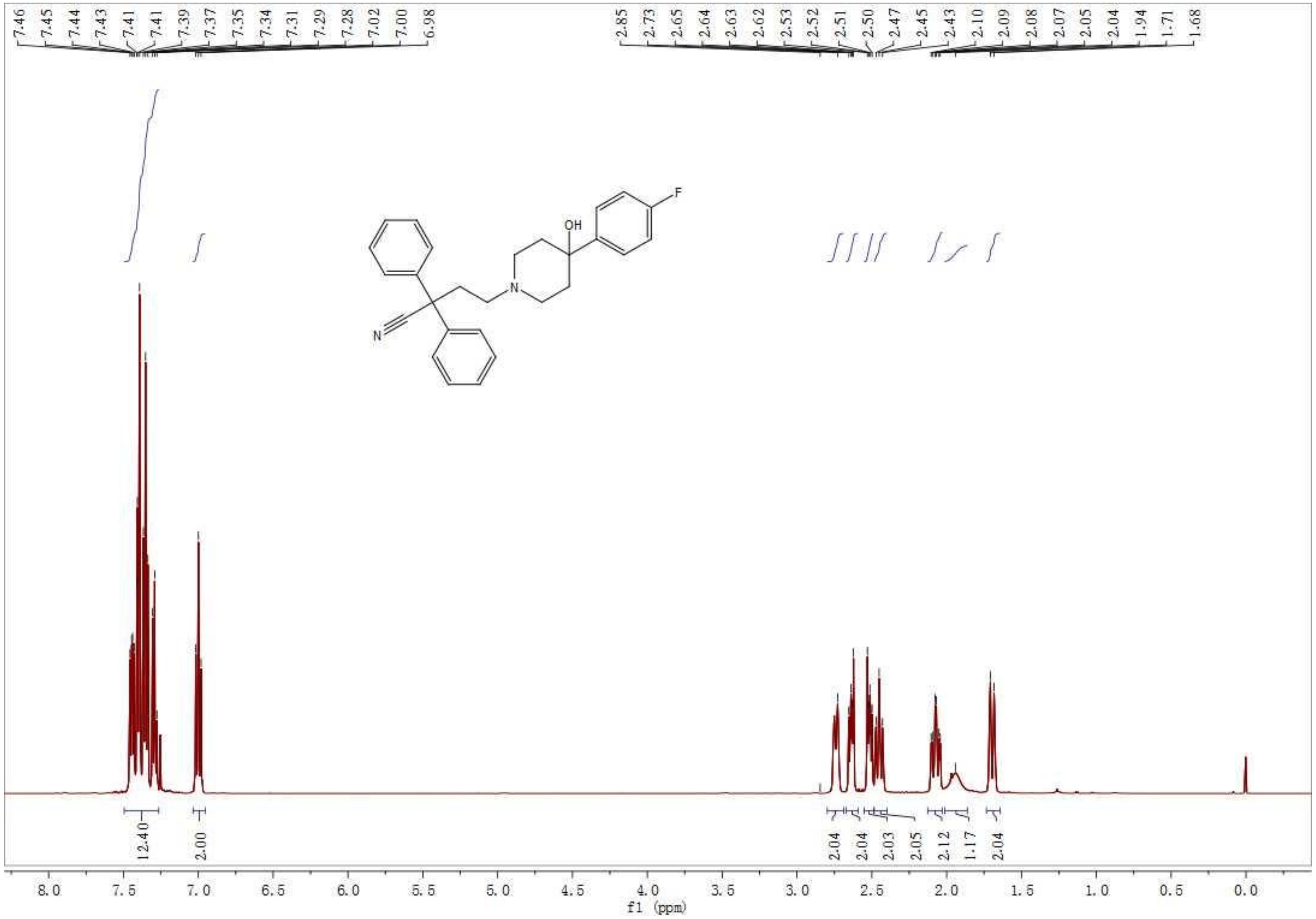

Figure S4. 125 MHz ^{13}C-NMR spectrum of Compound **12a**.

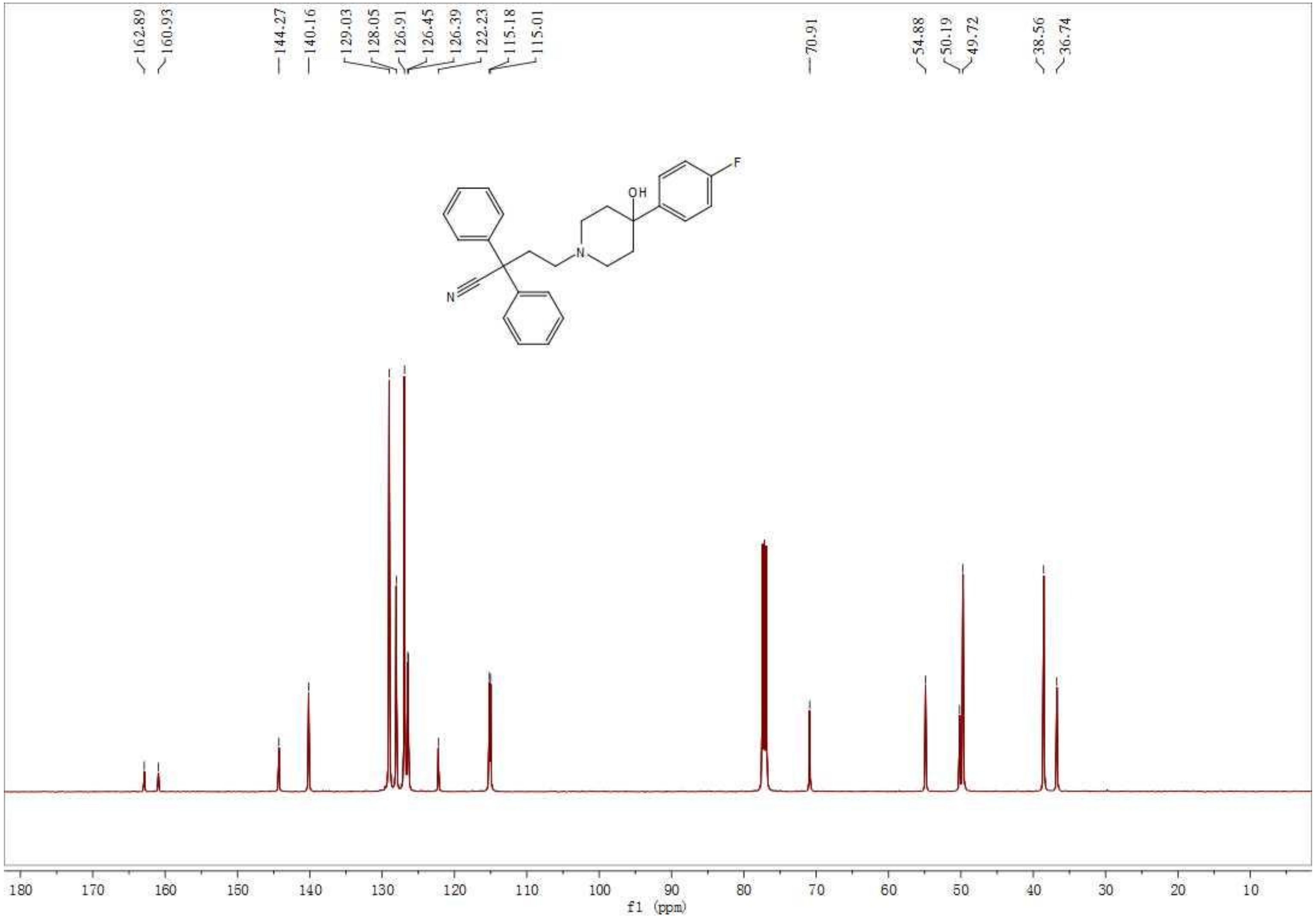

Figure S5. 470 MHz ^{19}F-NMR spectrum of Compound **12a**.

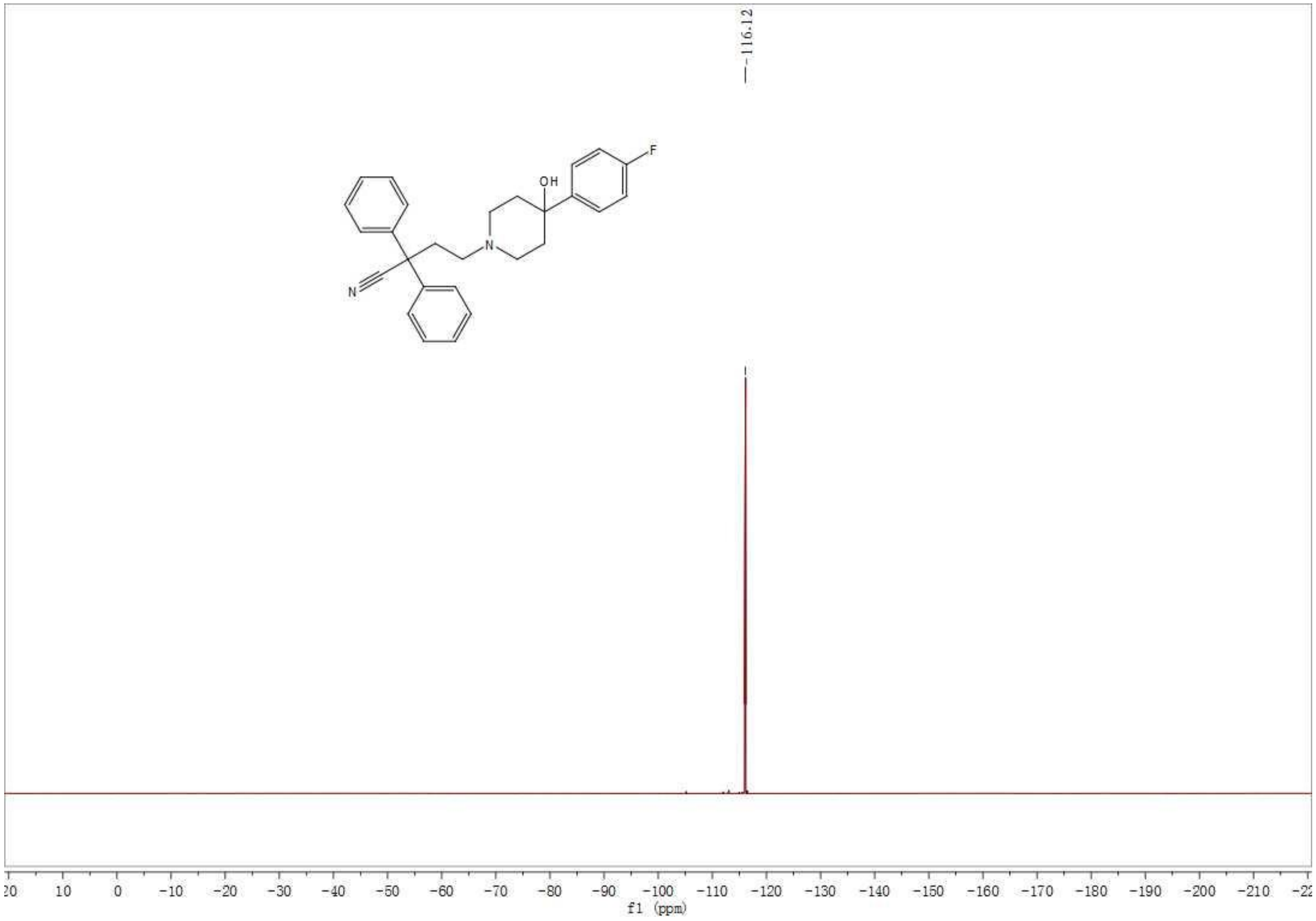

Figure S6. MS spectrum of Compound **12a**.

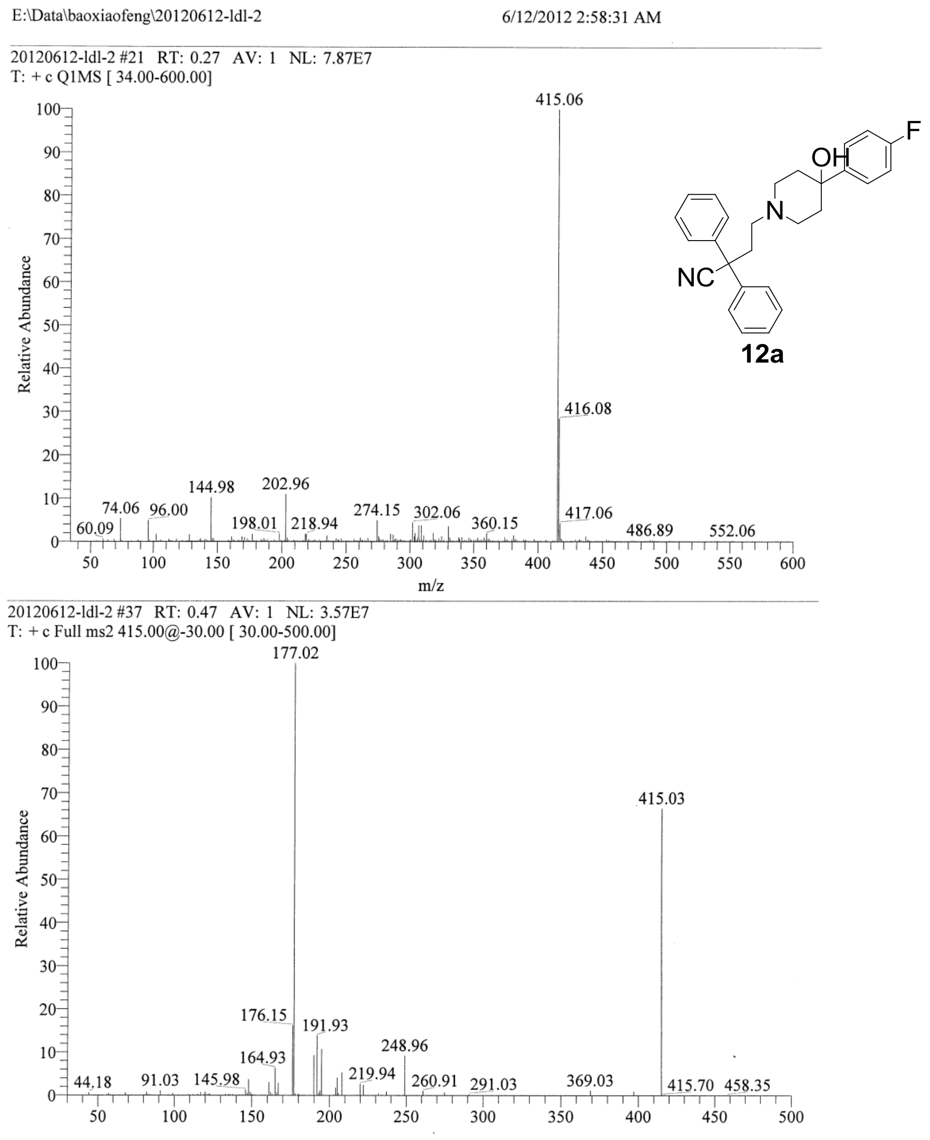

Figure S7. 500 MHz ^{1}H-NMR spectrum of Compound **12b**.

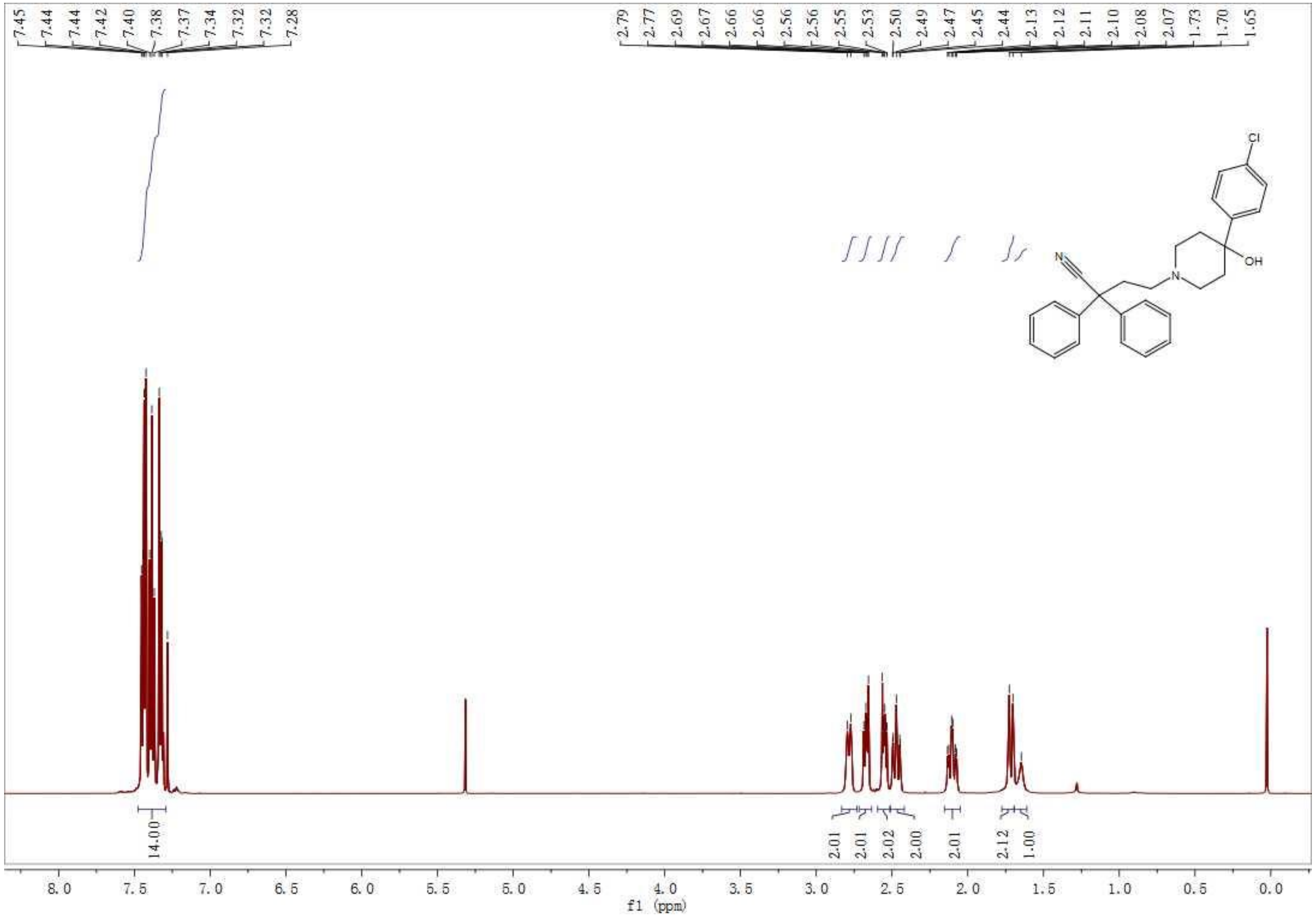

Figure S8. 500 MHz ^{1}H-NMR spectrum of Compound **12c**.

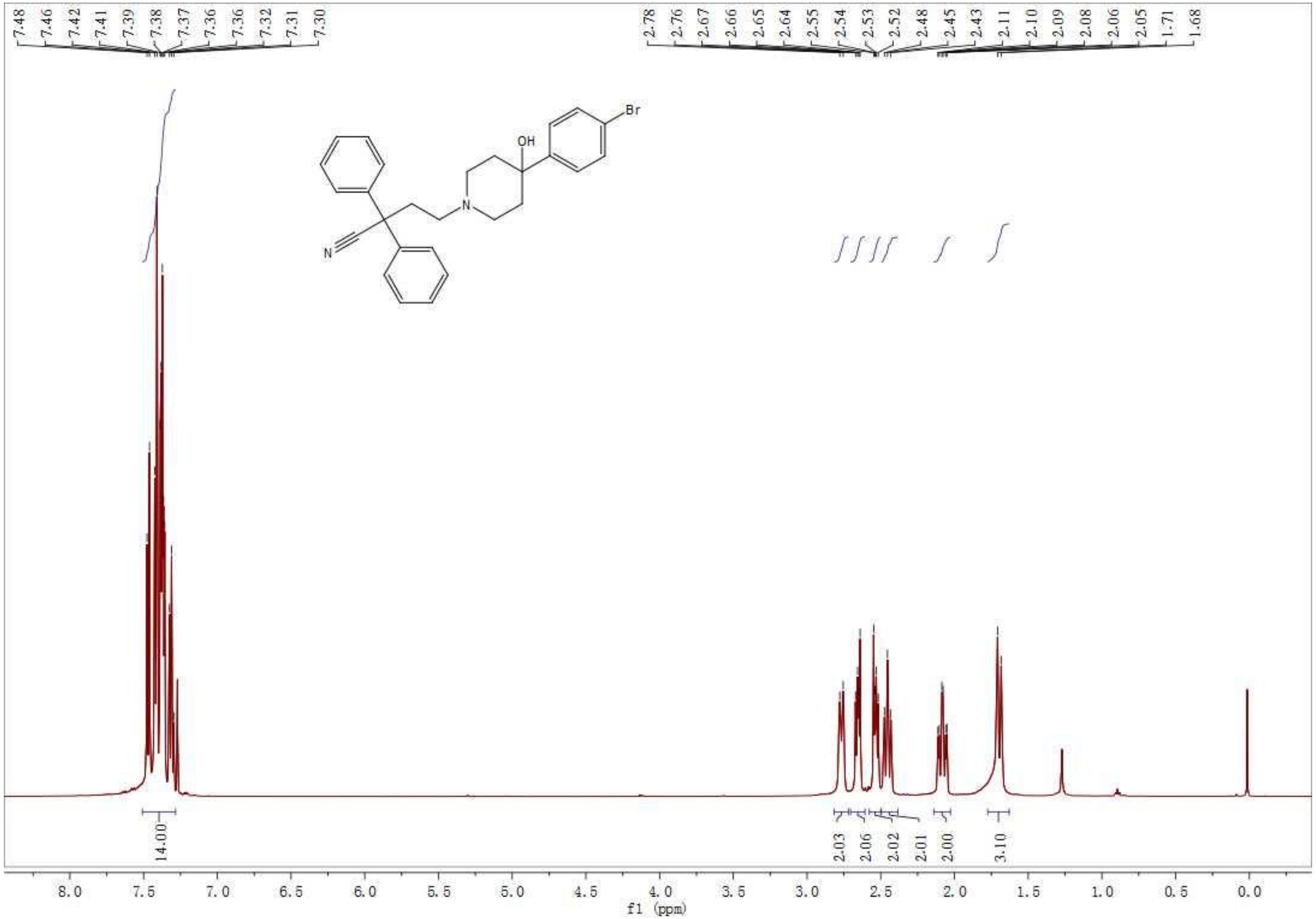

Figure S9. 125 MHz ^{13}C-NMR spectrum of Compound **12c**.

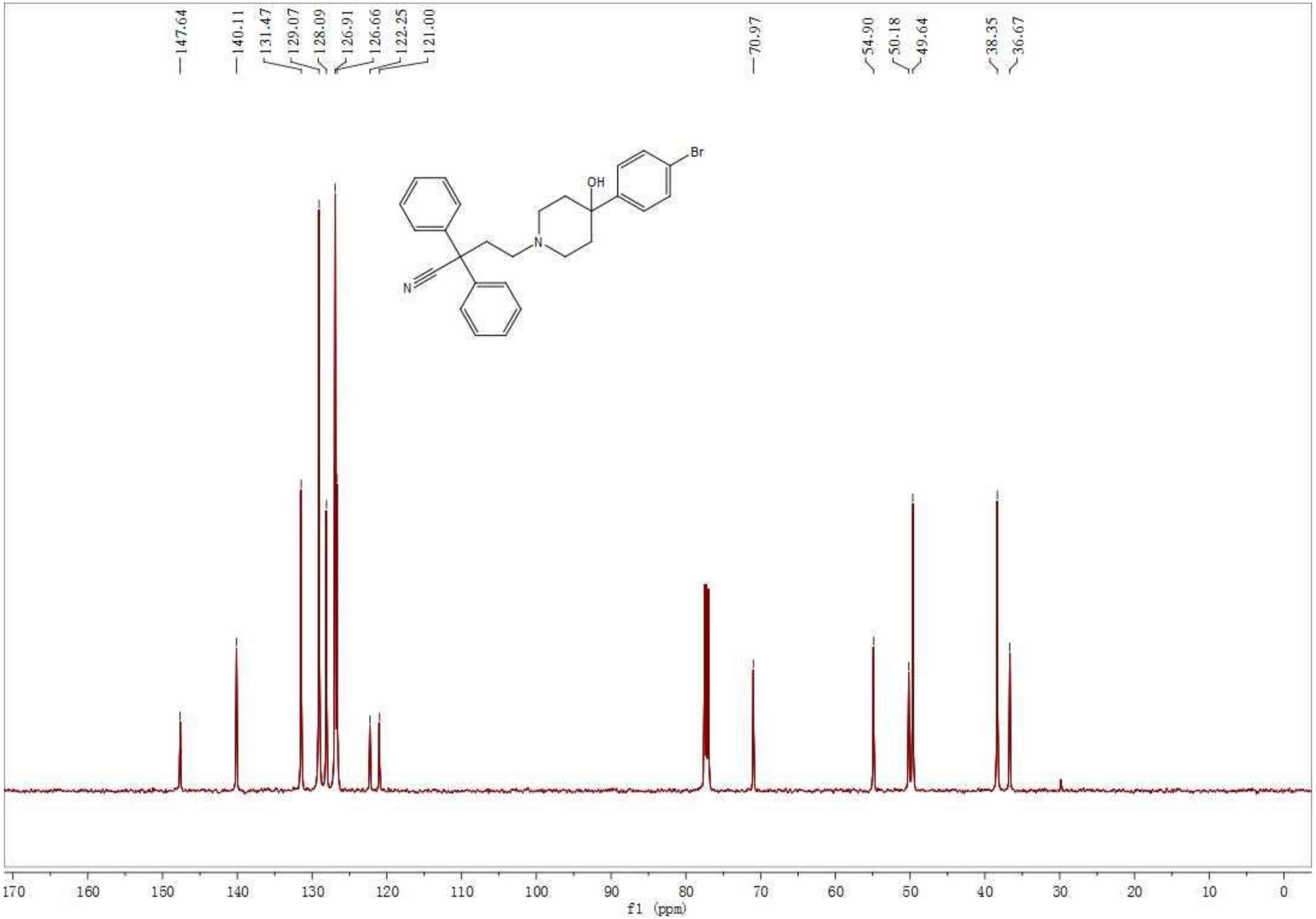

Figure S10. MS spectrum of Compound **12c**.

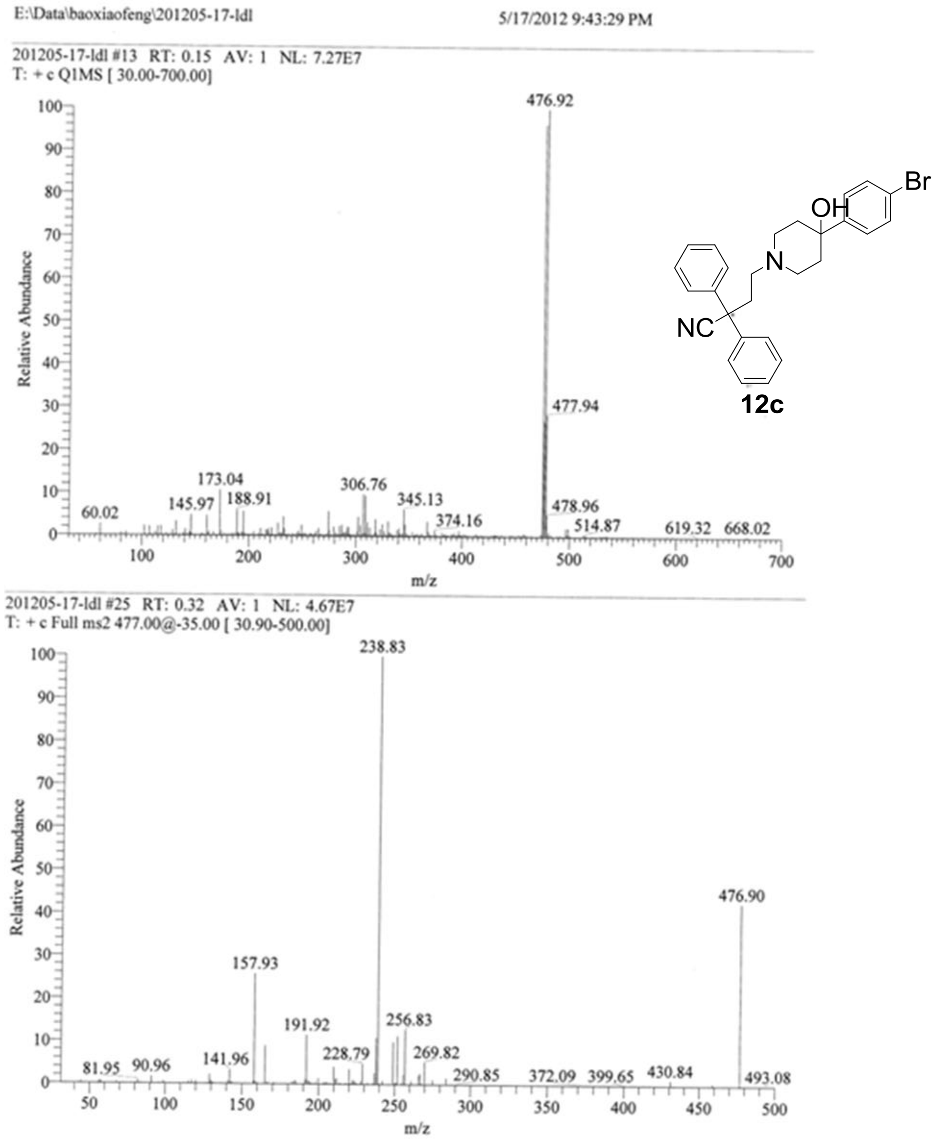

Figure S11. 500MHz ^{1}H-NMR spectrum of Compound **6**.

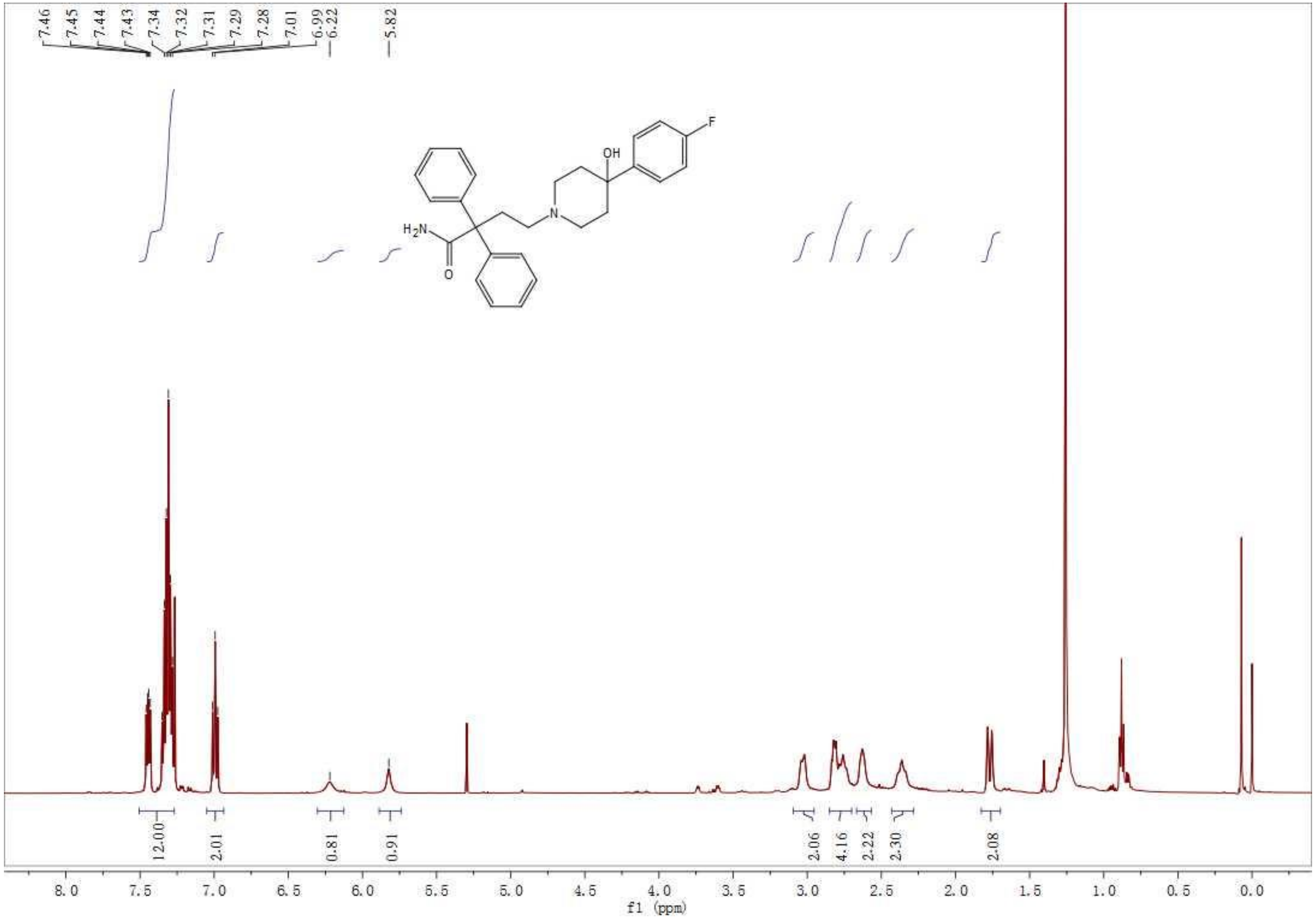

Figure S12. 125 MHz ^{13}C-NMR spectrum of Compound **6**.

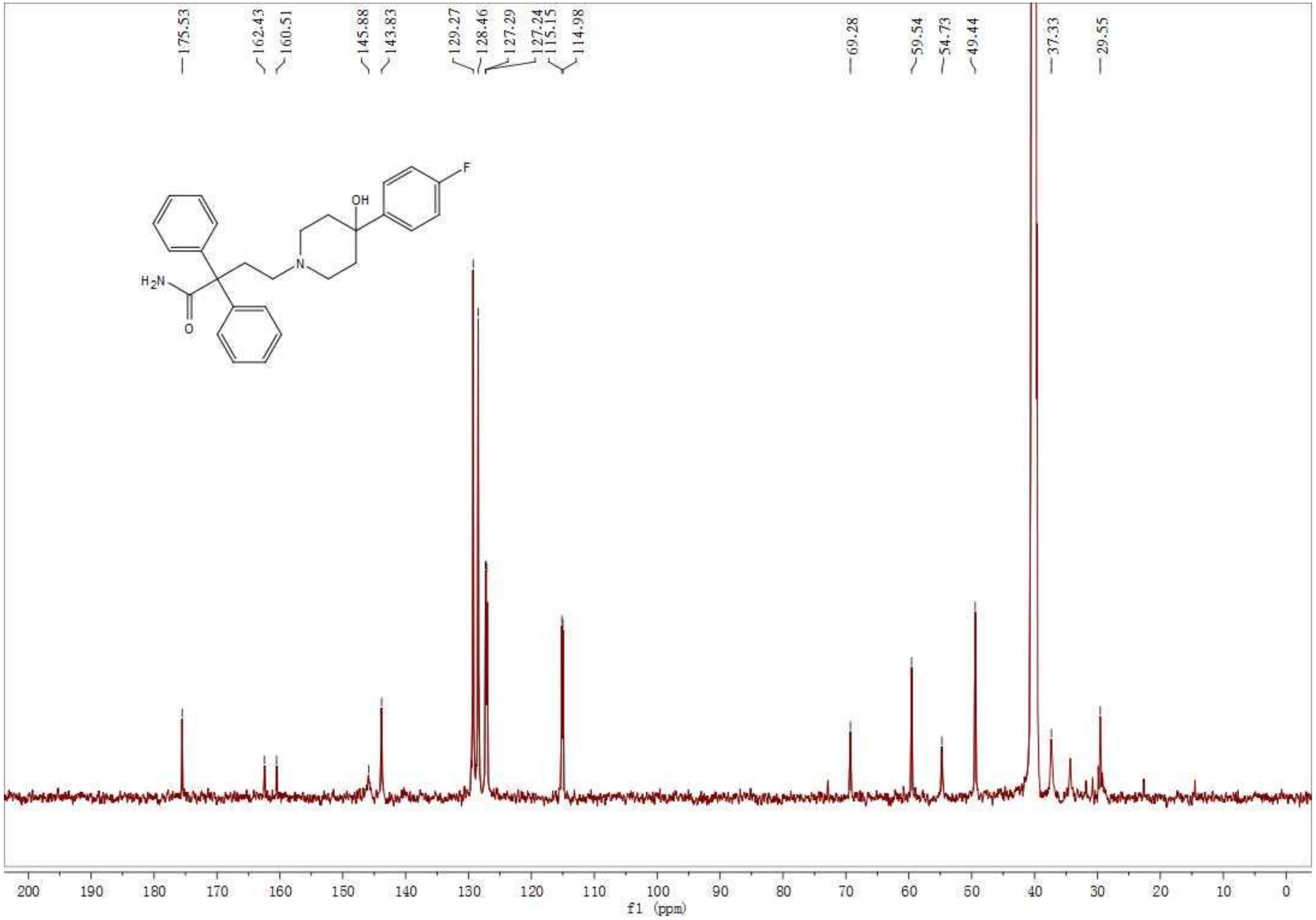

Figure S13. 470 MHz ^{19}F-NMR spectrum of Compound **6**.

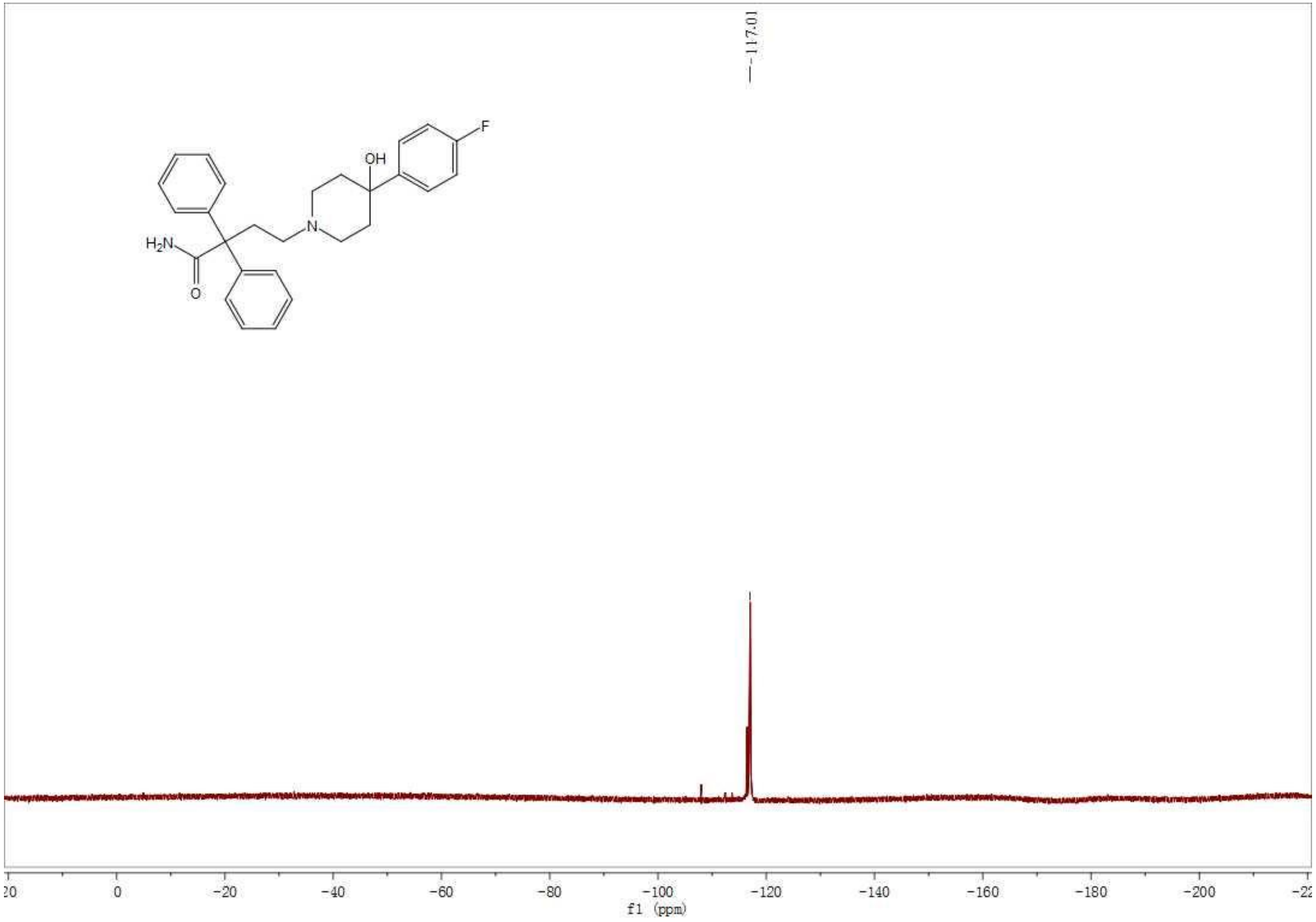

Figure S14. 500MHz ^{1}H-NMR spectrum of Compound **13b**.

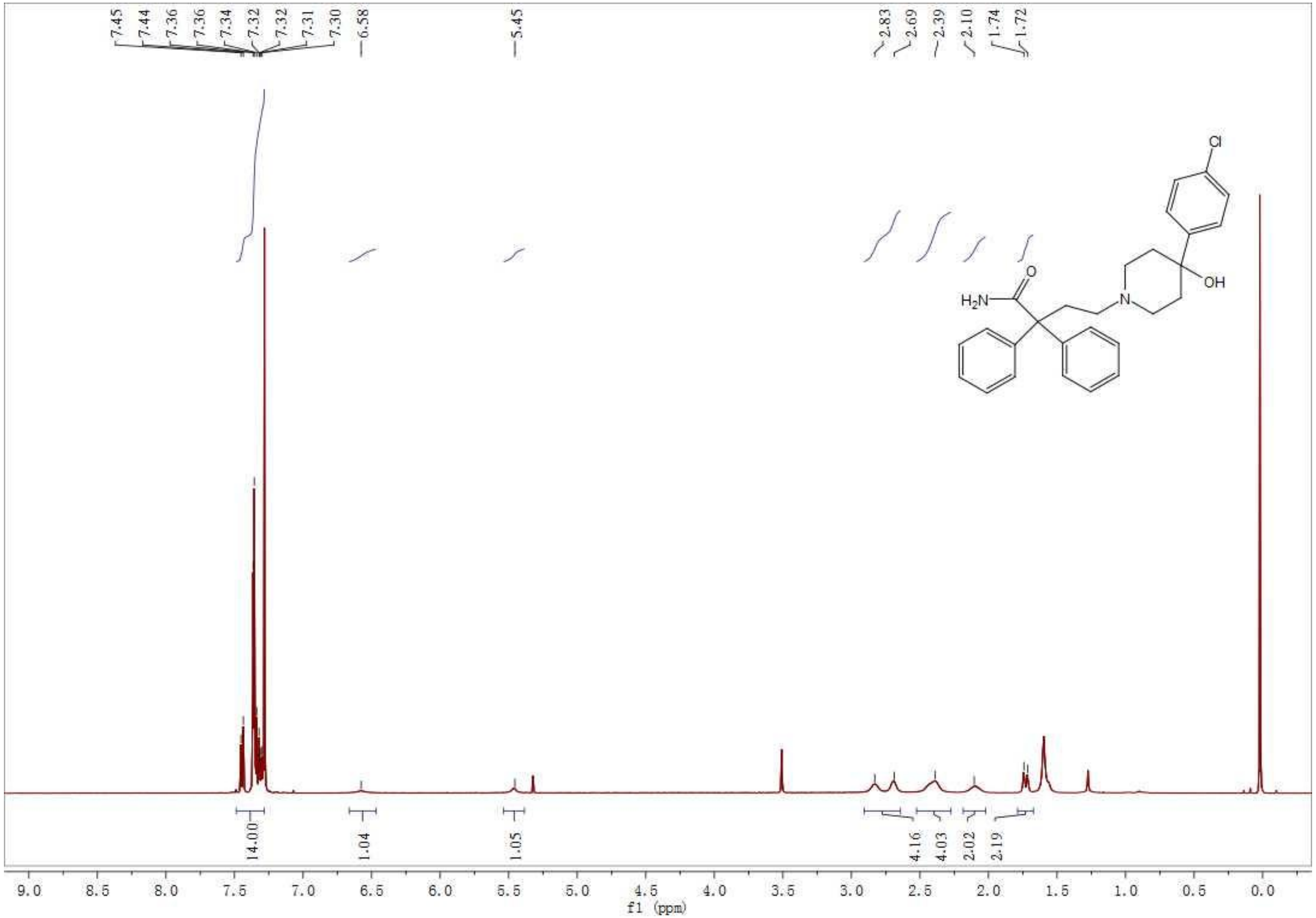

Figure S15. 100 MHz ^{13}C-NMR spectrum of Compound **13b**.

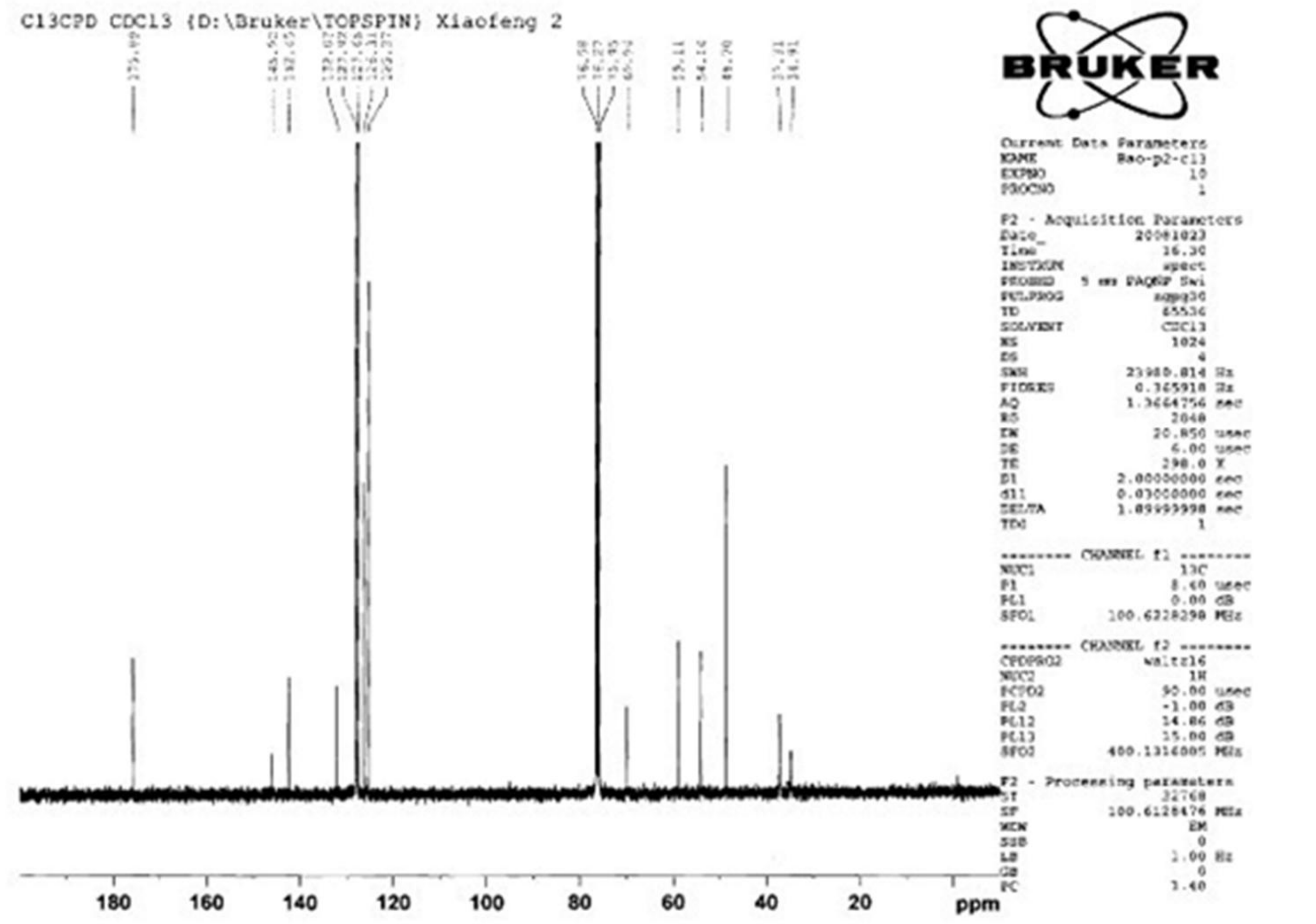

Figure S16. LC-MS spectrum of Compound **13b**.

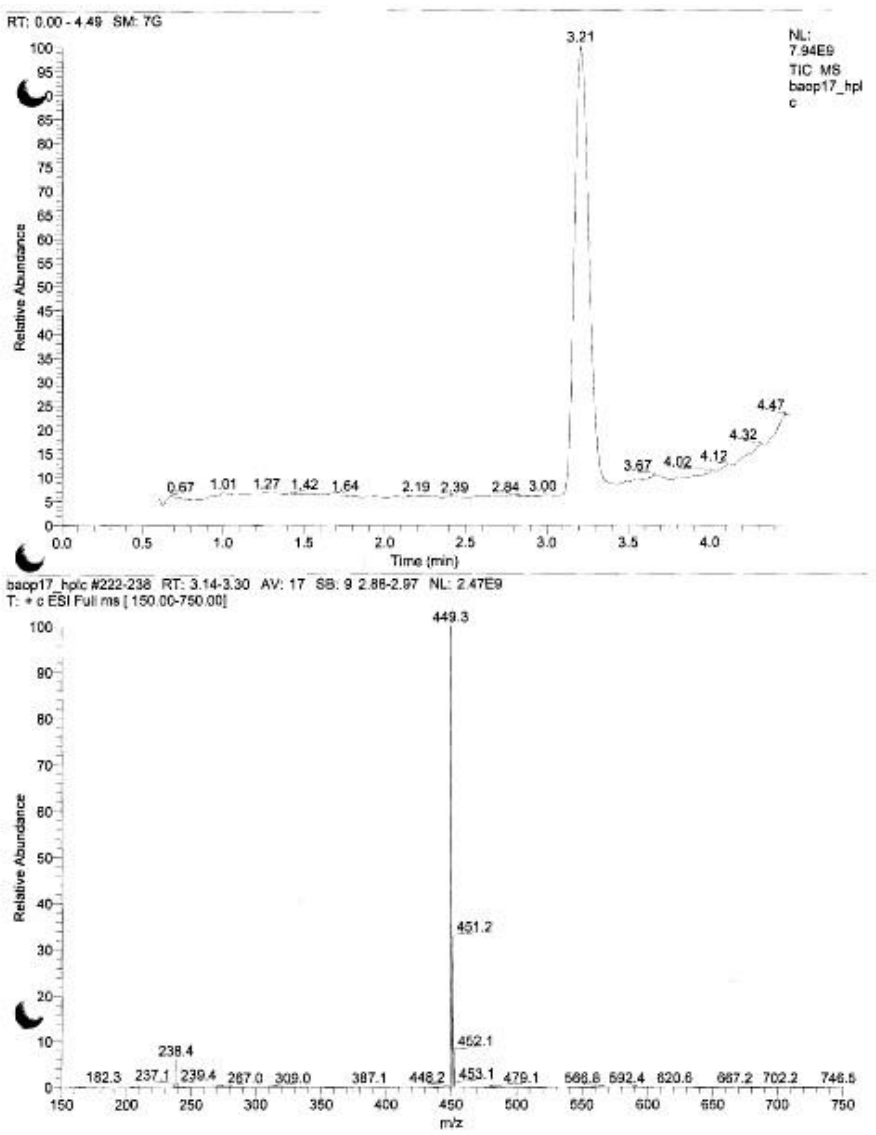

Figure S17. 500 MHz ^{1}H-NMR spectrum of Compound **7.**

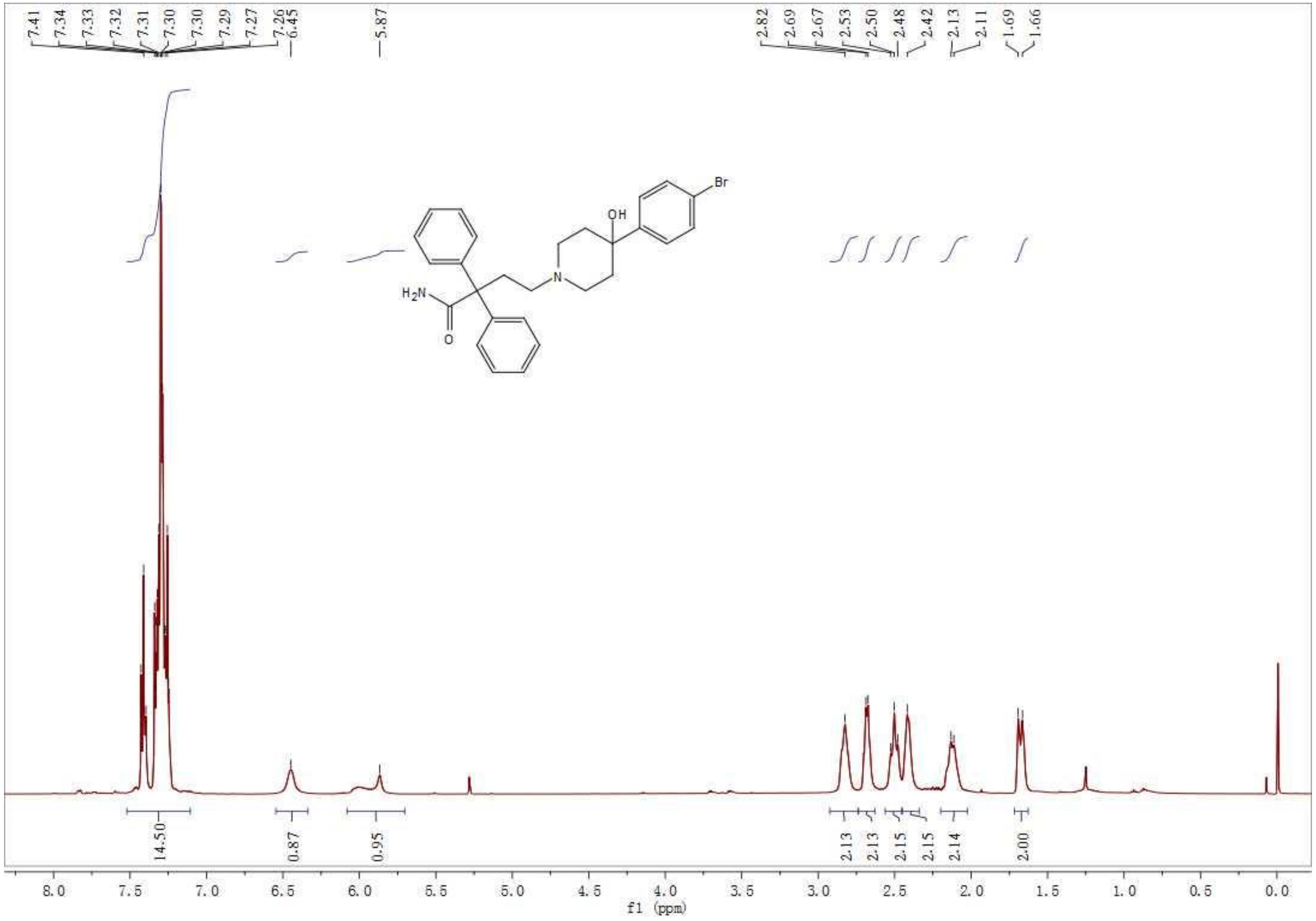

Figure S18. 125 MHz ^{13}C-NMR spectrum of Compound **7**.

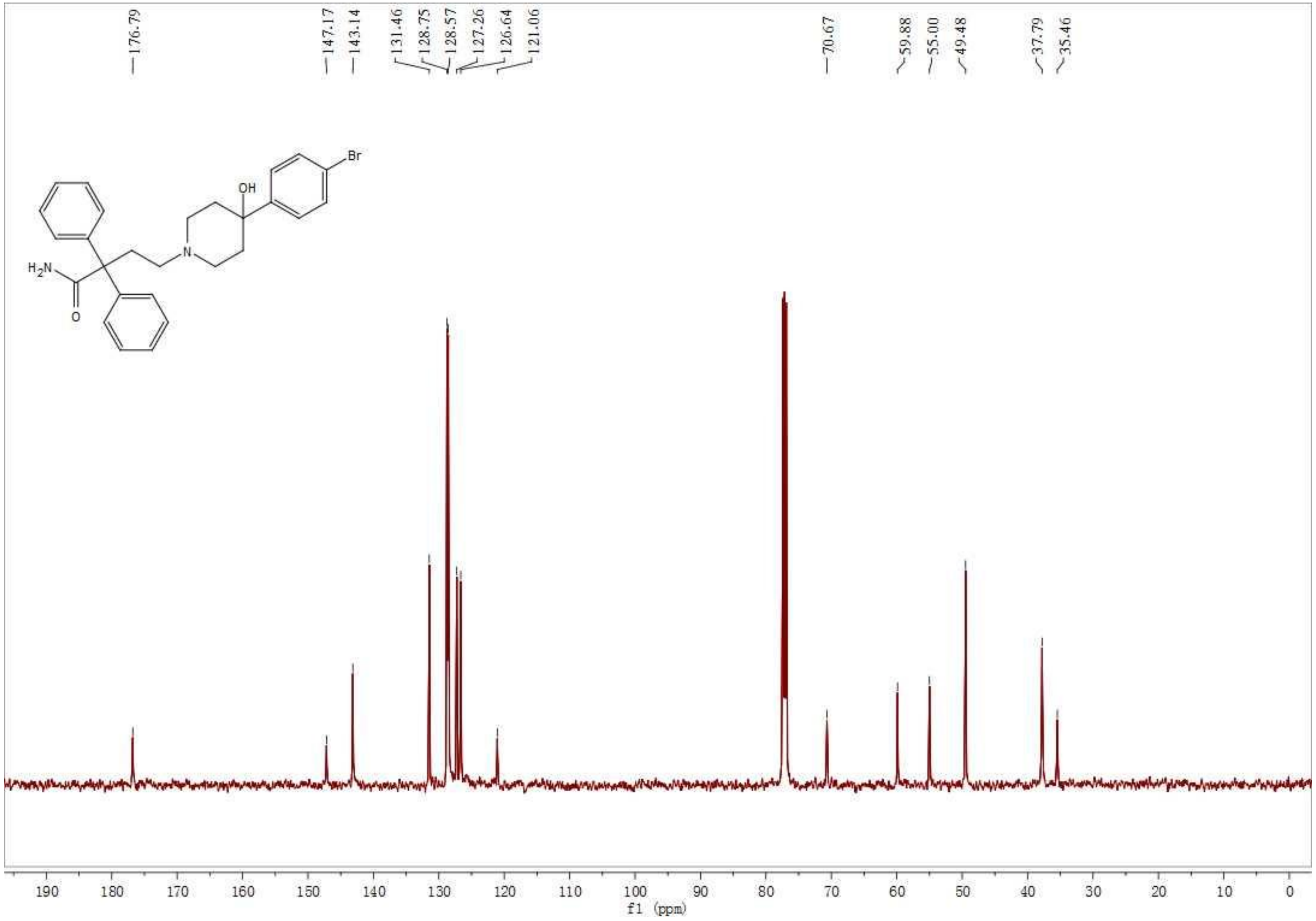

Figure S19. MS spectrum of Compound **7**.

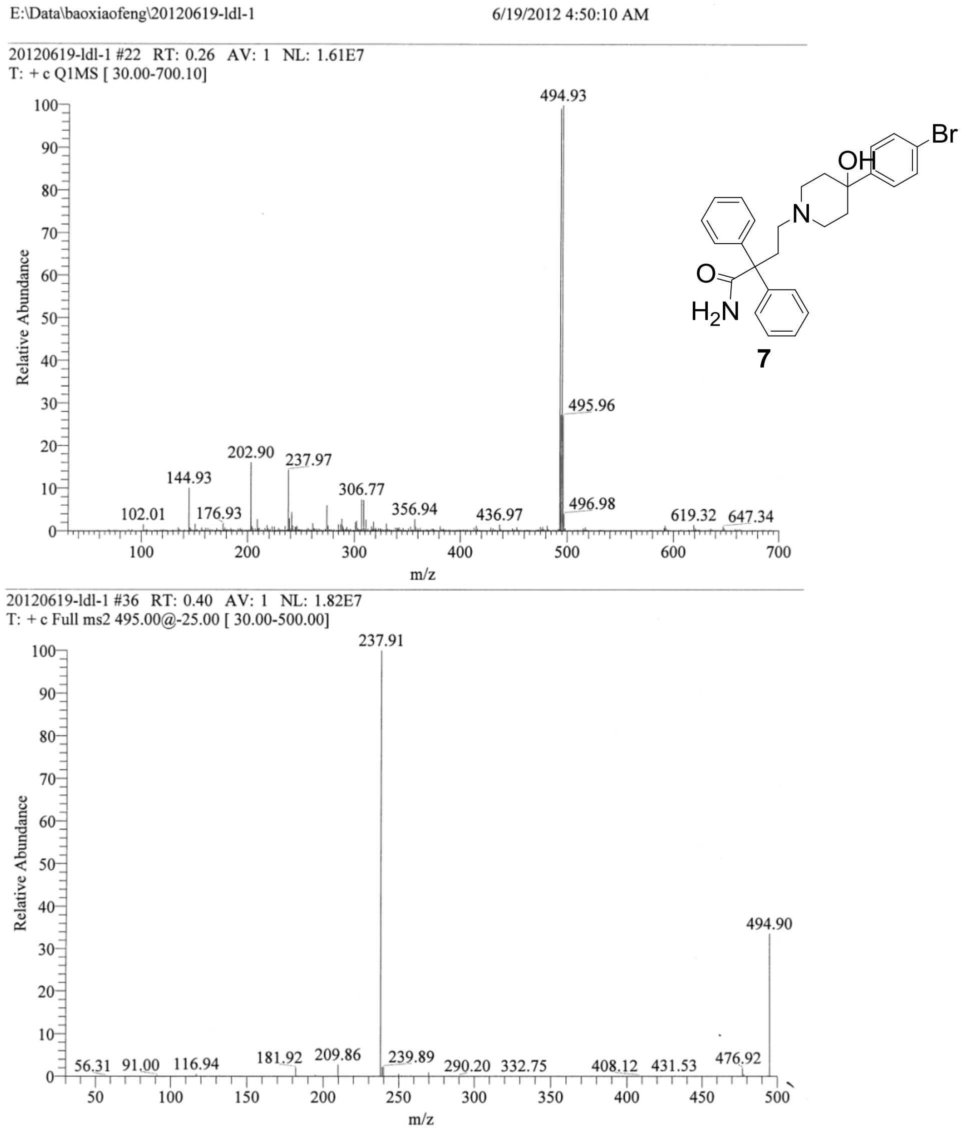

Figure S20. 500 MHz ¹H-NMR spectrum of Compound **14b**.

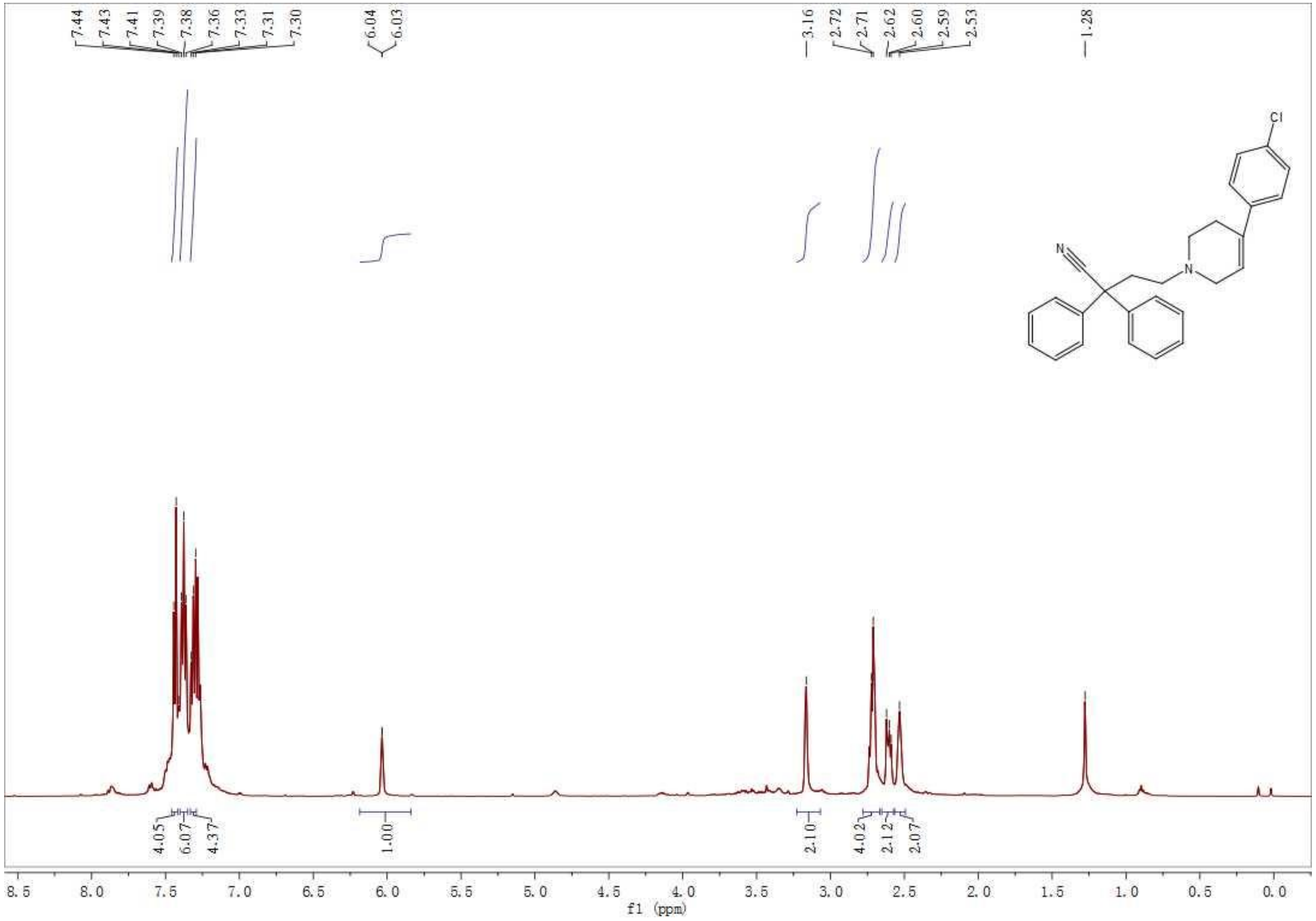

Figure S21. 125 MHz ^{13}C-NMR spectrum of Compound **14b**.

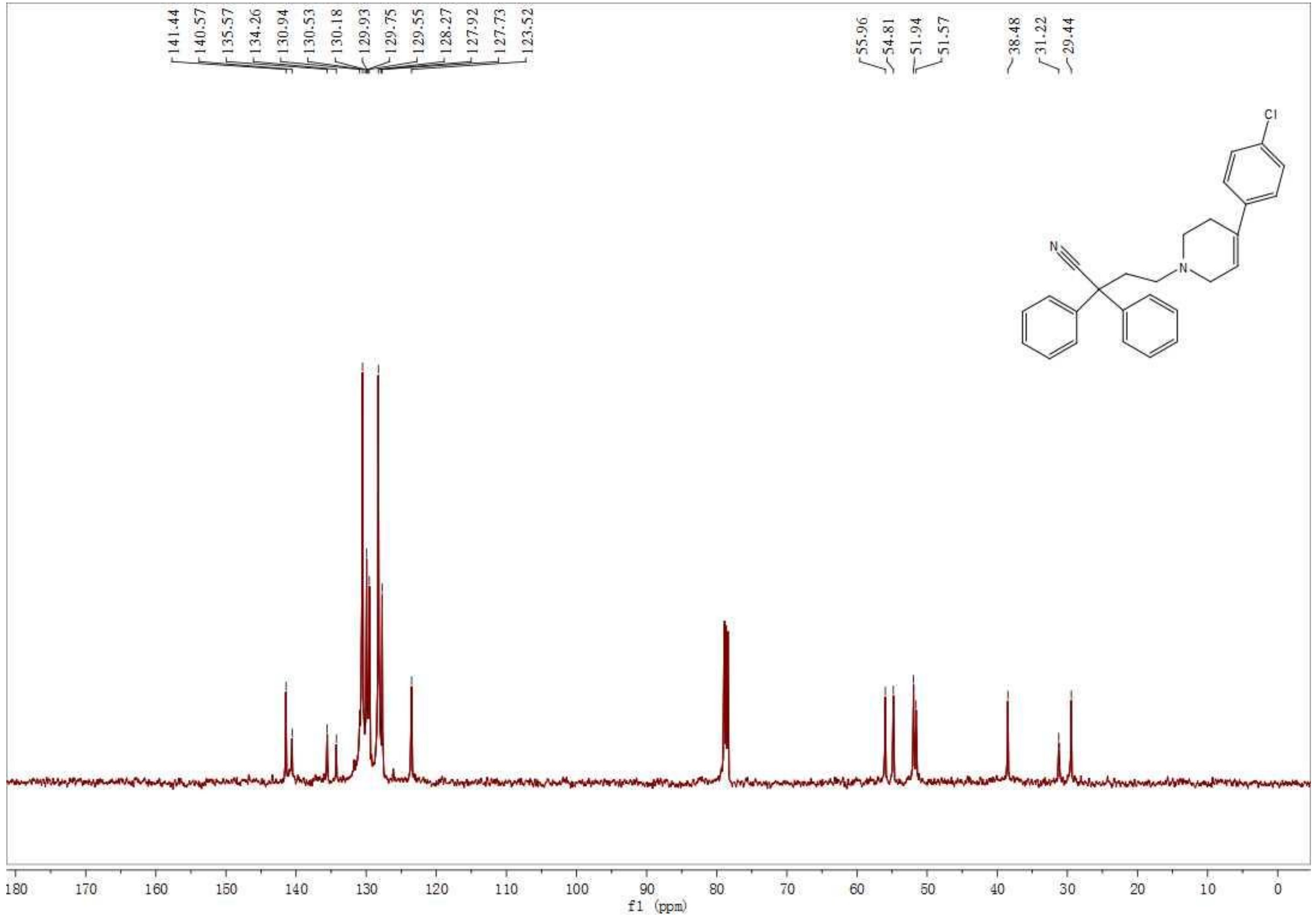

Figure S22. MS spectrum of Compound **14b**.

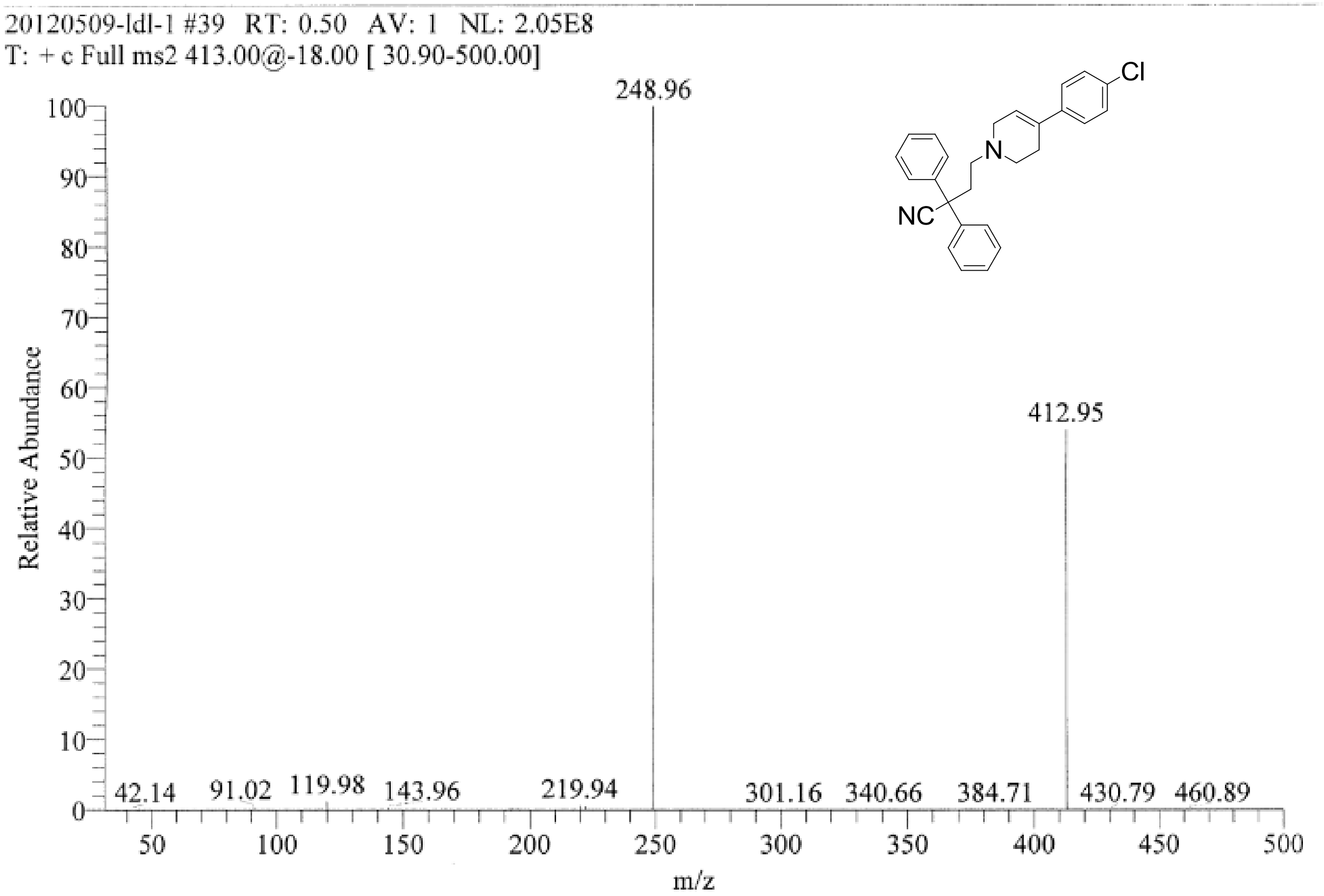

Figure S23. 500 MHz ^{1}H-NMR spectrum of Compound **18**.

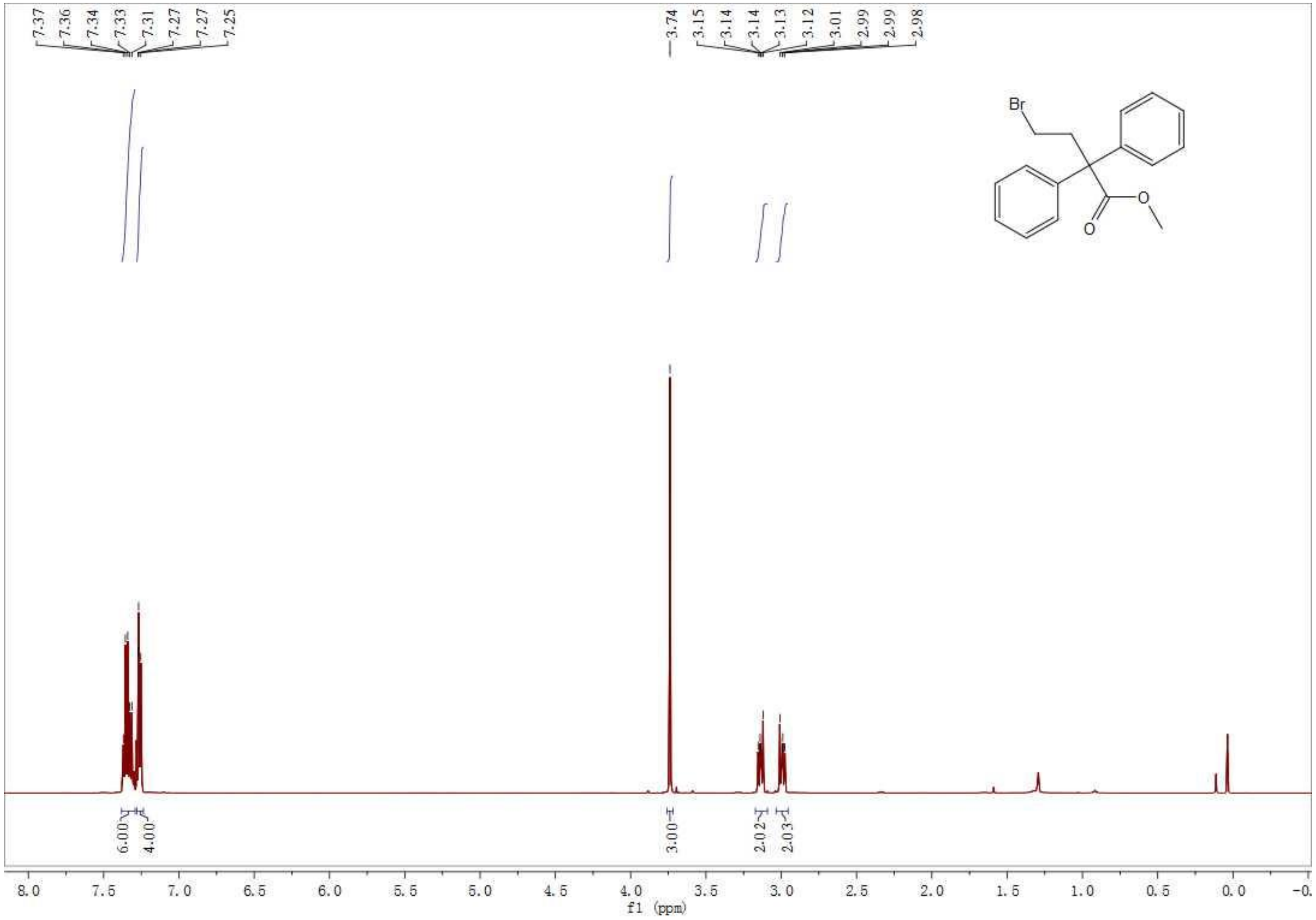

Figure S24. 125 MHz ^{13}C-NMR spectrum of Compound **18**.

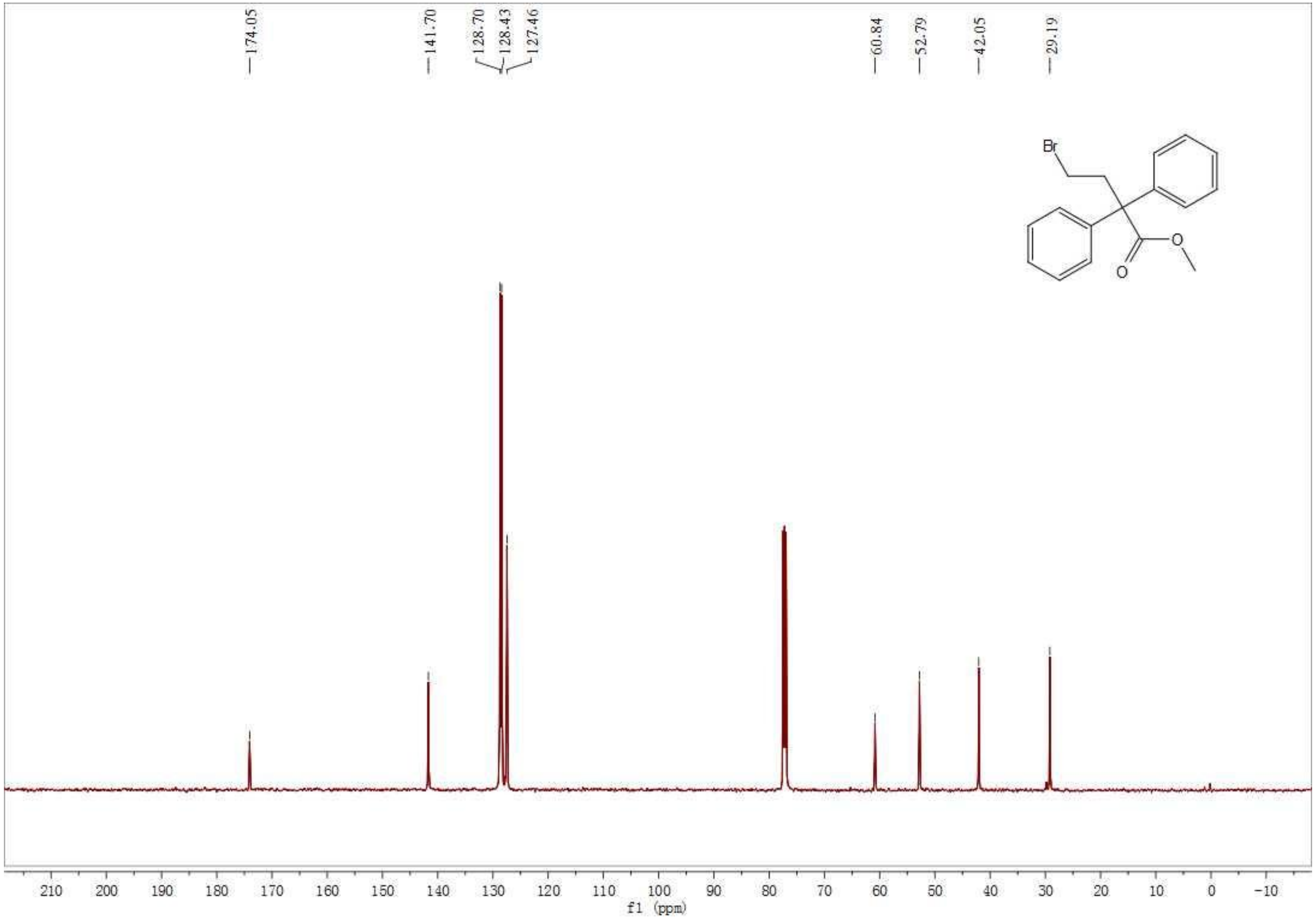

Figure S25. MS spectrum of Compound **18**.

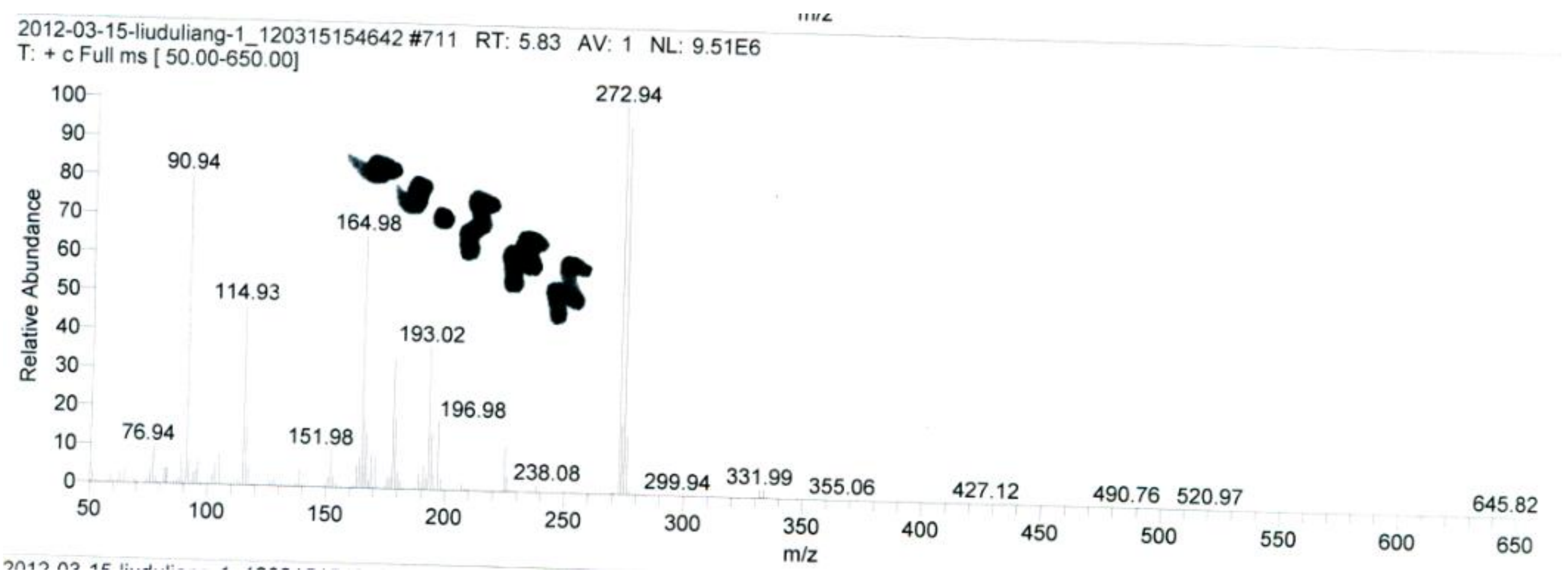

Figure S26. 500 MHz ^{1}H-NMR spectrum of Compound **19**.

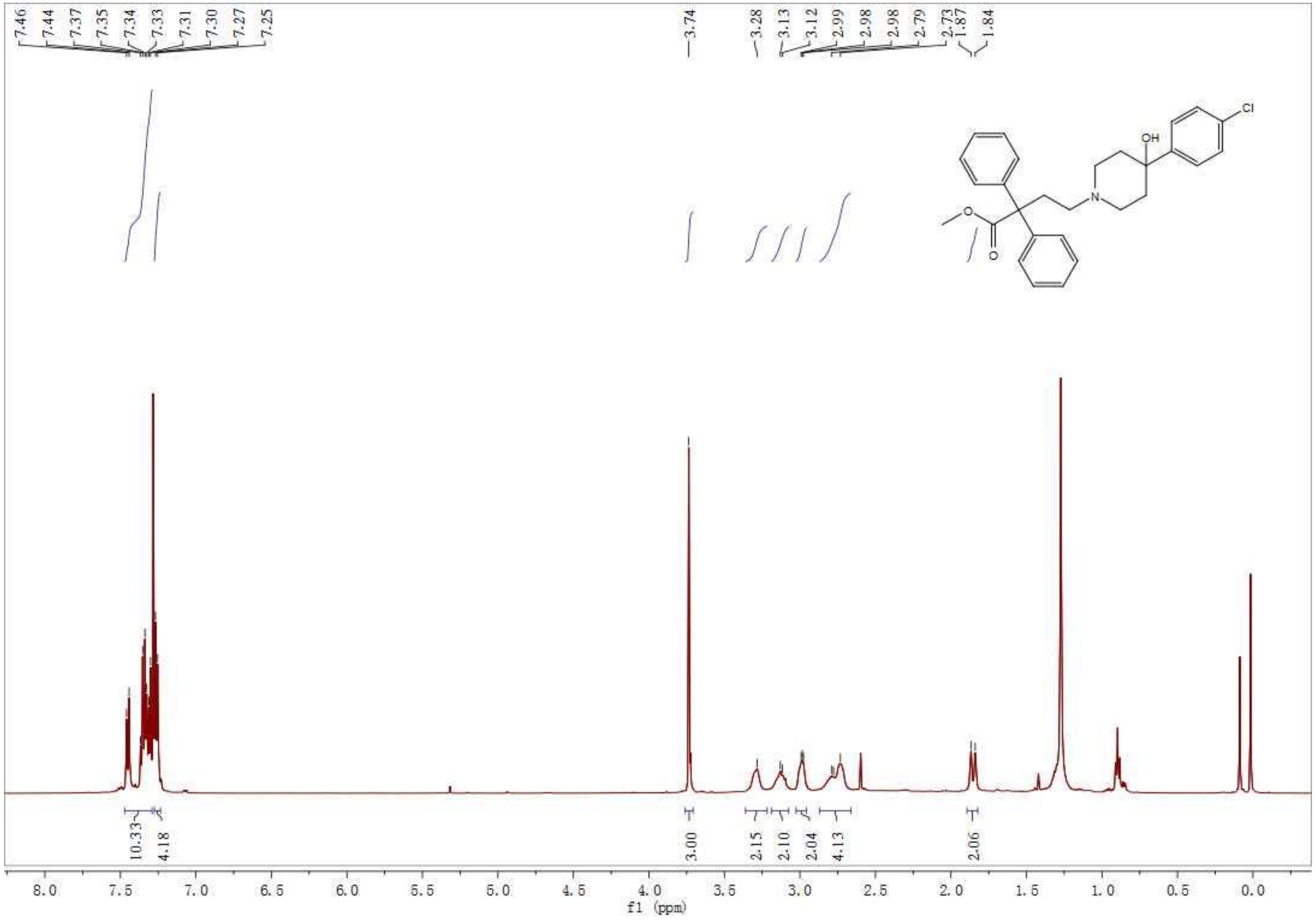

Figure S27. 125 MHz ^{13}C-NMR spectrum of Compound **19**.

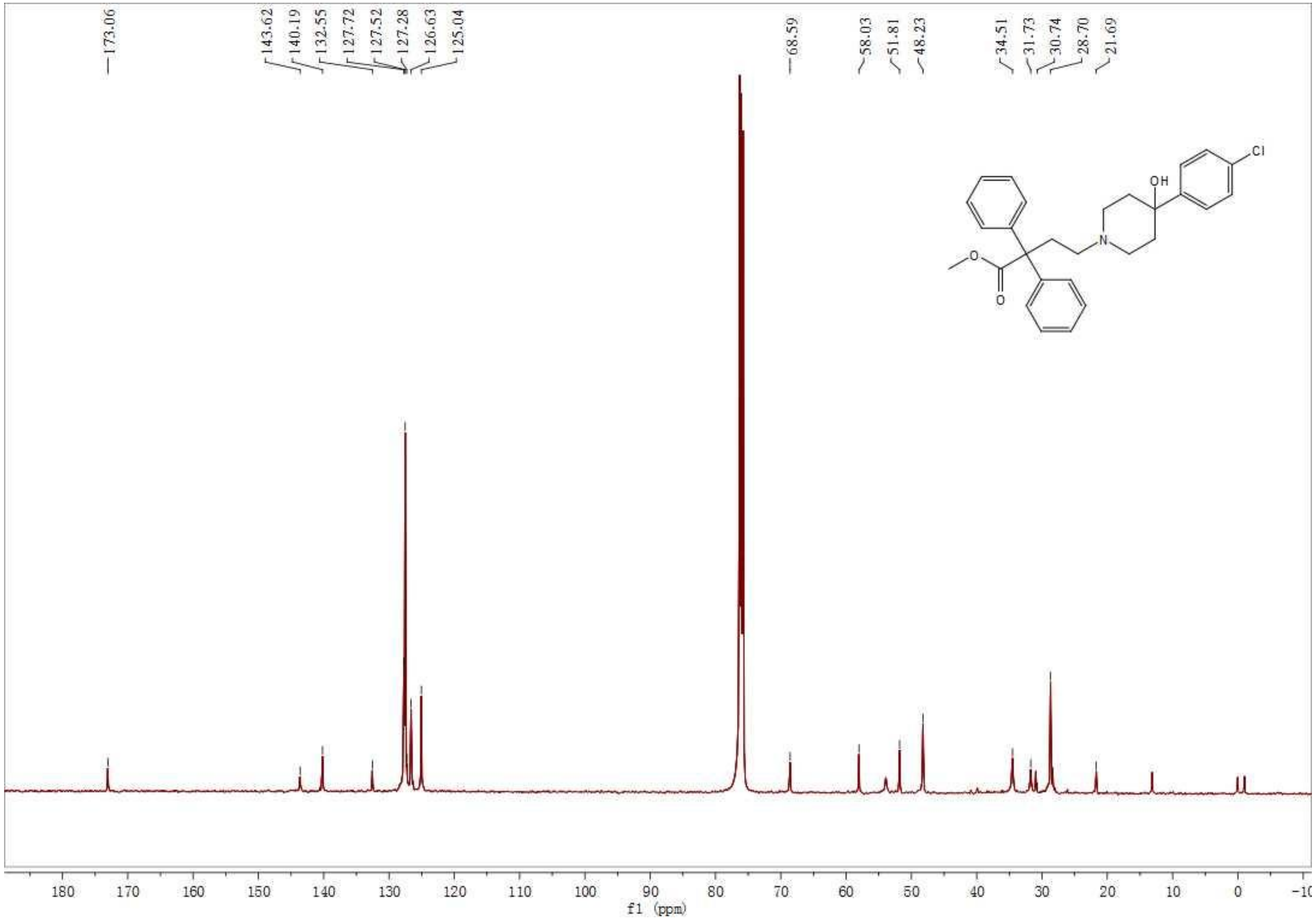

Figure S28. MS spectrum of Compound **19**.

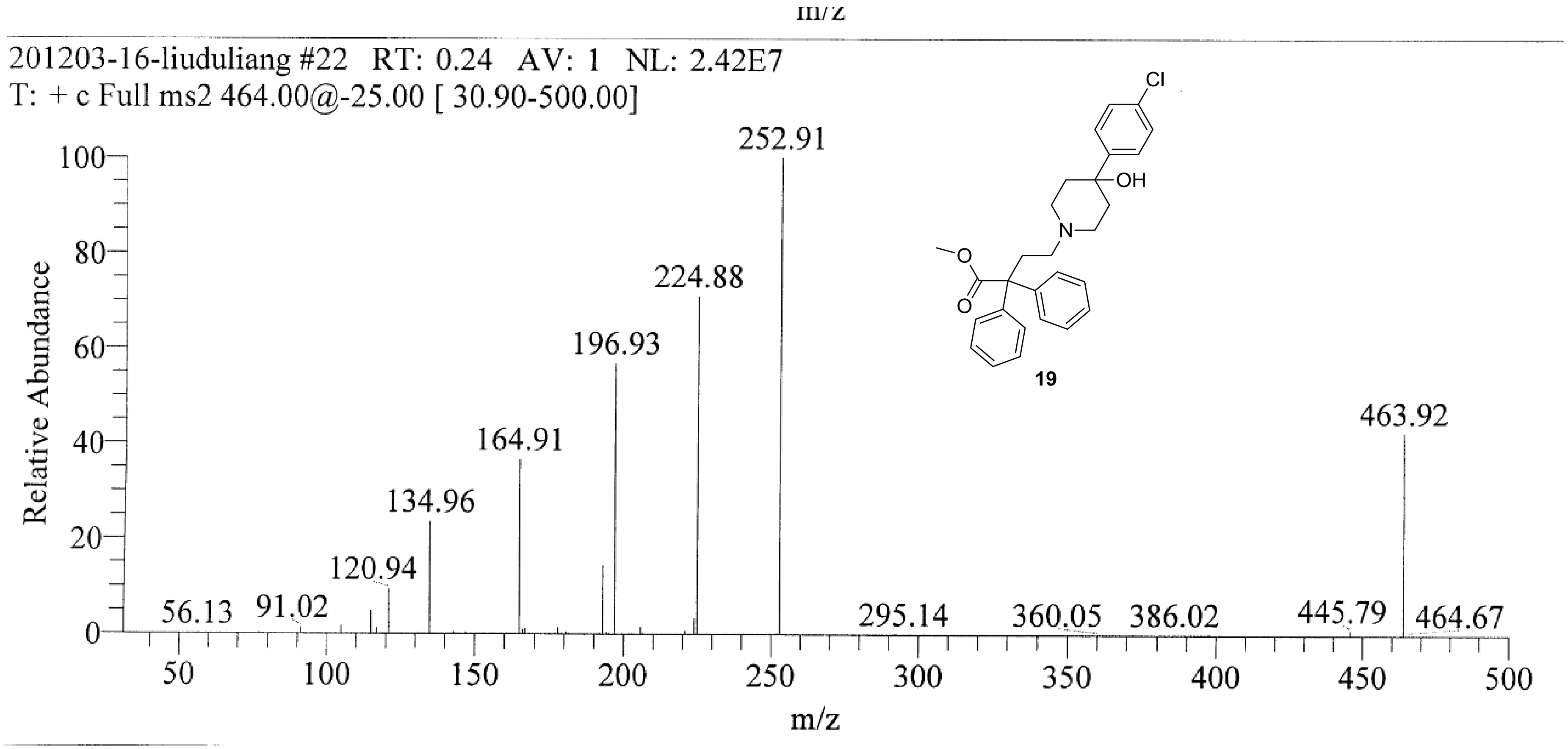

Figure S29. 500 MHz ^{1}H-NMR spectrum of Compound **5**.

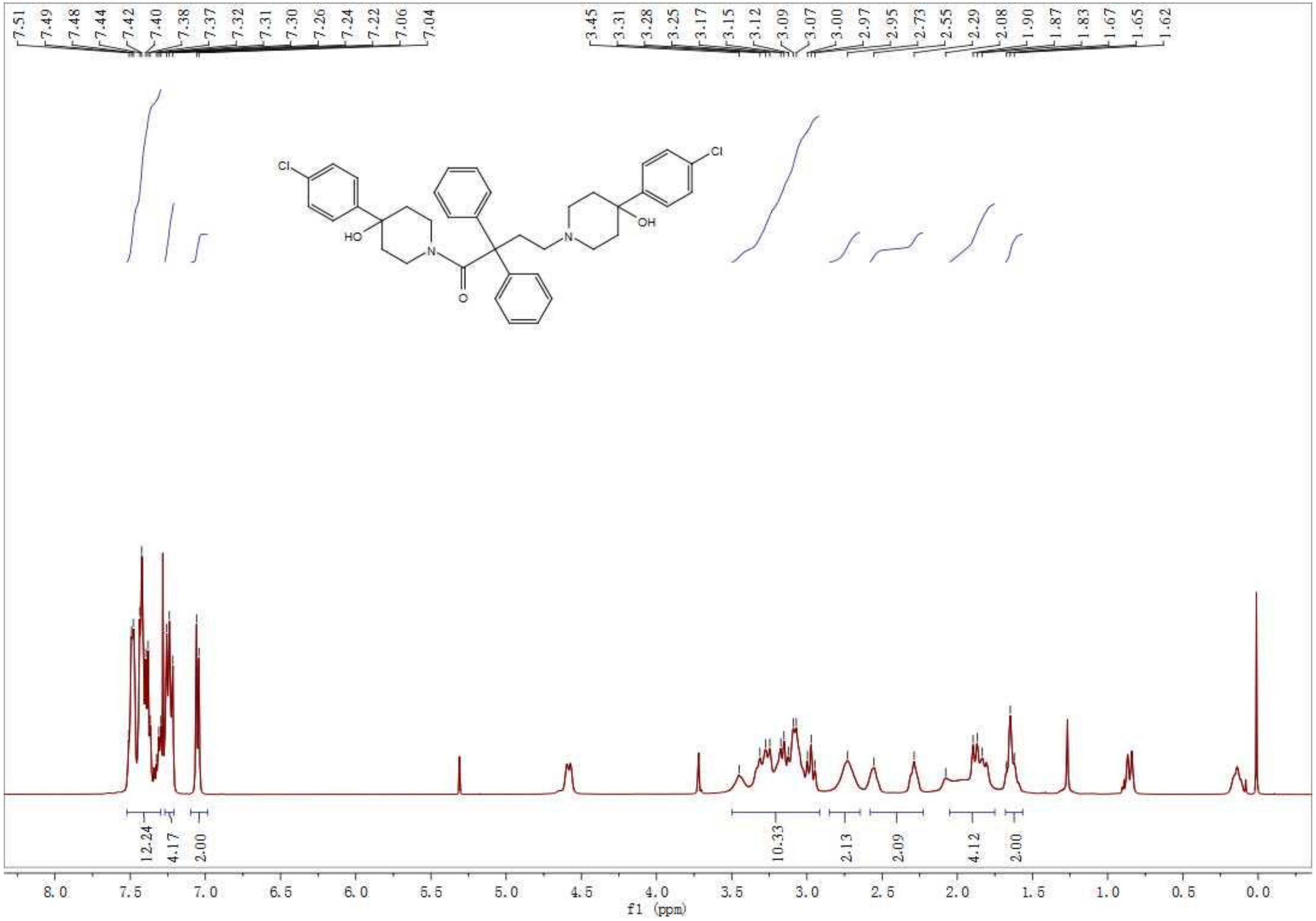

Figure S30. 125 MHz ^{13}C-NMR spectrum of Compound **5**.

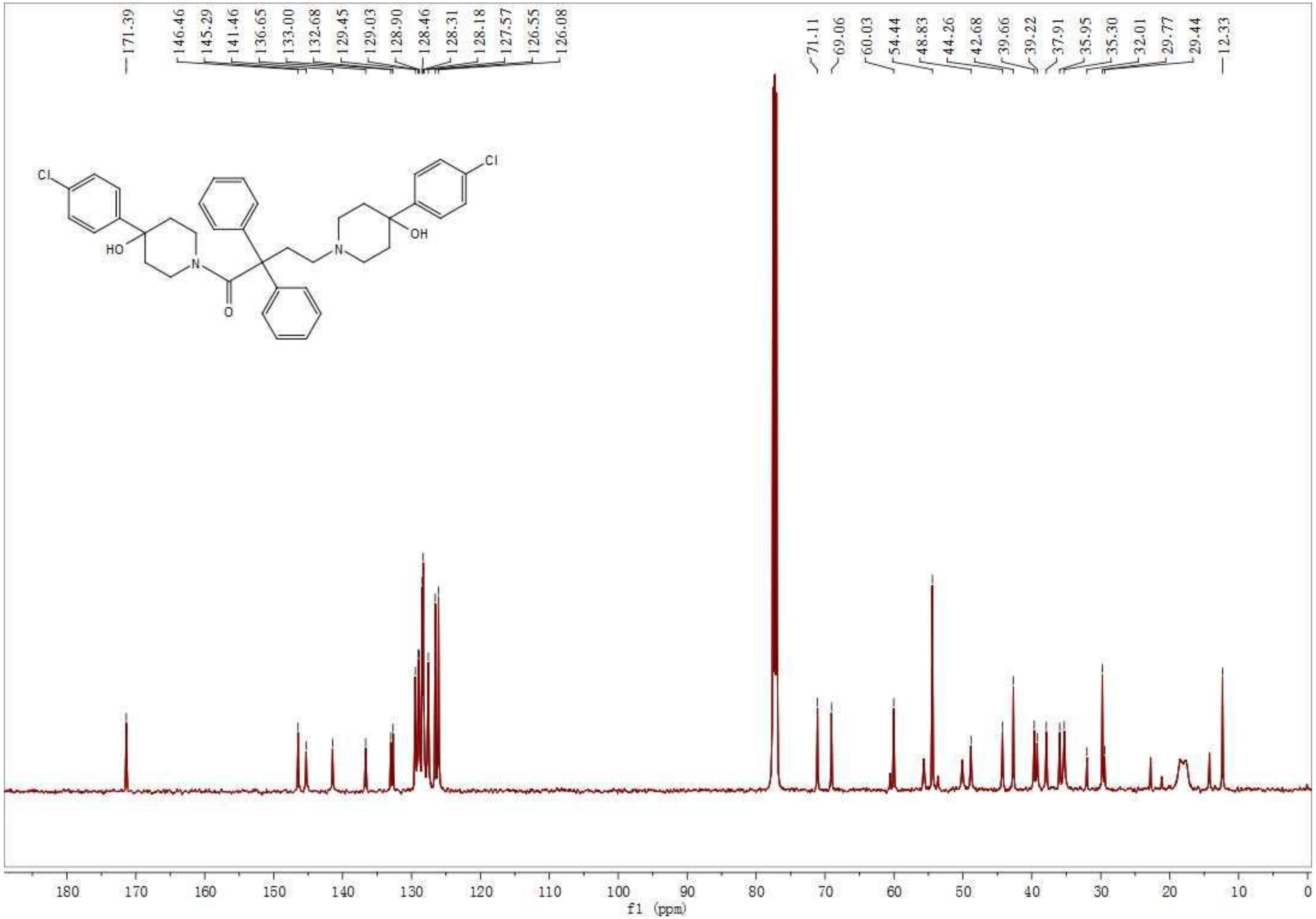

Figure S31. MS spectrum of Compound **5**.

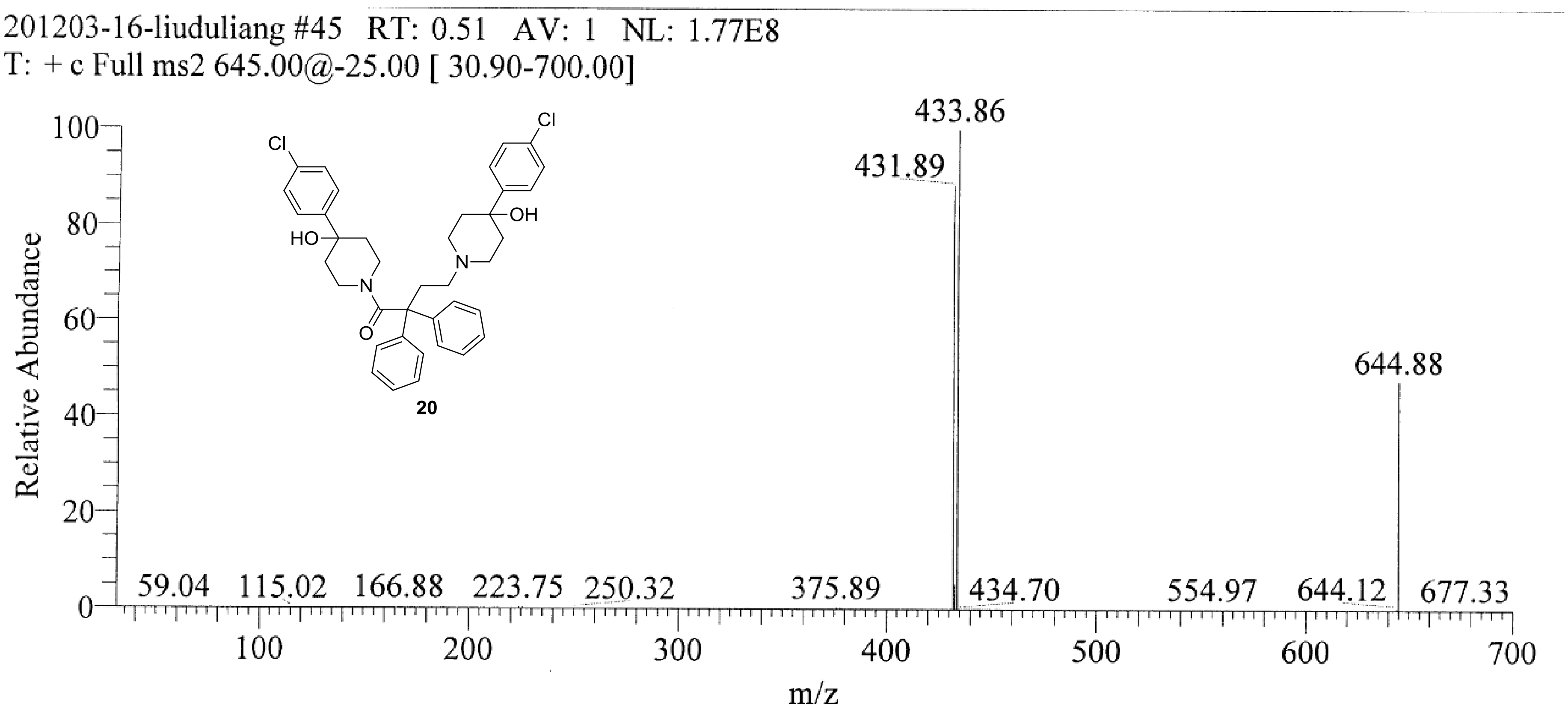

Supplement: Supplementary file 1 [file molecules-17-14288-s001.pdf]
